# Whole‐Epiphysis Trabecular Bone in Tamarin Limbs Suggests Effects of Leaping Distance Alongside Non‐Biomechanical Factors

**DOI:** 10.1002/ajpa.70293

**Published:** 2026-06-08

**Authors:** Uyen Nguyen, Fabio Alfieri, Alessio Veneziano, Annika Licht, John A. Nyakatura

**Affiliations:** ^1^ Comparative Zoology, Institute of Biology, Humboldt‐Universität zu Berlin Berlin Germany; ^2^ Institute of Ecology and Evolution, Universität Bern Bern Switzerland; ^3^ Department of Earth Sciences University of Cambridge Cambridge UK; ^4^ Museum Für Naturkunde, Leibniz‐Institut Für Evolutions‐ Und Biodiversitätsforschung Berlin Germany; ^5^ Archéozoologie, Archéobotanique: Sociétés, Pratiques et Environnements (AASPE), Muséum National D'Histoire Naturelle, CNRS Paris France

**Keywords:** bone functional adaptation, cancellous bone, *Leontocebus*, primate locomotion, *Saguinus*

## Abstract

**Objectives:**

Beyond non‐biomechanical factors, the trabecular architecture of long bone epiphyses variably underlies functional adaptations to locomotor behavior. A recent study, characterizing tamarin trabecular bone using traditional metrics derived from Volumes of Interest (VOIs), identified a preliminary relationship with leaping distance. We hypothesized that quantifying trabecular topological indices from whole epiphyses in the same system would enhance detection of locomotor signals.

**Materials and Methods:**

We analyzed μCT scans of humeral and tibial whole‐epiphyseal trabecular bone in four tamarin species representing short‐ and long‐distance leapers. We quantified topological indices—node density (NodDen), trabecular tortuosity (TrabTort), trabecular length (TrabLen), and fractal dimension (FD)—alongside traditional metrics—degree of anisotropy (DA) and bone volume fraction (BV/TV). We tested relationships between trabecular traits and leaping distance and assessed the effects of non‐functional factors (sex and captivity).

**Results:**

Long‐distance leapers exhibited significantly higher NodDen in humeral and tibial epiphyses and increased distal humeral TrabTort. Regions of elevated NodDen were concentrated beneath the humeroscapular joint (proximal humerus) and occurred variably across other epiphyses. In contrast, DA, BV/TV, TrabLen, and FD were more strongly associated with sex and captivity.

**Discussion:**

Higher loading magnitudes associated with longer leaps may increase NodDen and, more tentatively, TrabTort in tamarin limb epiphyses. However, locomotion‐related signals were weaker than those linked to sex and captivity, suggesting that leaping distance affects specific trabecular traits, whereas the overall trabecular organization is driven by non‐functional factors. Whole‐epiphysis analyses and topological indices provide a valuable complement to traditional VOI‐based approaches and metrics.

## 1 Introduction

Leaping requires anatomical adaptations that generate high power over short intervals (Mo et al. 2020). In primates, it has been widely studied from a morpho‐functional perspective (e.g., Ryan and Ketcham 2001, 2002, 2005; Ryan and Van Rietbergen 2005). For instance, leaping primates exhibit robust, elongated femora with enlarged heads, characterized by more preferentially aligned (anisotropic) trabeculae and higher trabecular bone volume fraction (BV/TV) (Connour et al. 2000; Polvadore et al. 2024; Ryan and Ketcham 2001). Because force production varies among primates with different leaping behaviors (Demes et al. 1996, 1999), anatomical adaptations may differ not only between leaping and non‐leaping taxa but also among species employing distinct leaping strategies.

Recently, Berles et al. (2024) examined *Saguinus midas* , *S. mystax* , *S. imperator* , and *Leontocebus nigrifrons* , showing that these tamarins differ in the distance of leaping, a behavior that accompanies their arboreal quadrupedalism. *L. nigrifrons* and *S. mystax* leap farther than *S. imperator* and *S. midas* (hereafter “long leapers” and “short leapers,” respectively). In that work, long bone morphology—assessed using external and internal anatomy, including proximal/distal humeral and femoral trabecular structure—did not segregate species by behavior. Yet, long leapers showed proximal femoral trabeculae that were, albeit non‐significantly, more anisotropic—as indicated by higher degree of anisotropy (DA)—and also had higher BV/TV (Berles et al. 2024).

Trabecular bone structure adapts and/or evolves in response to locomotor behavior and the associated mechanical environment (Alfieri et al. 2022, 2023; Biewener et al. 1996; Kivell 2016), while also being influenced by non‐locomotor aspects as body mass (Doube et al. 2011; Alfieri, Demuth, et al. 2025), phylogeny (Ryan and Shaw 2013), sex (Eckstein et al. 2007), and captivity (Zack et al. 2022). Among trabecular parameters, DA and BV/TV are the most mechanically informative and commonly used (Arias‐Martorell et al. 2021; Maquer et al. 2015; Stauber et al. 2006; Ryan and Ketcham 2001). Locomotor behaviors involving more directionally stereotyped and/or higher‐magnitude loading are hypothesized to result in higher DA and/or BV/TV, respectively (Arias‐Martorell et al. 2021; Harrigan and Mann 1984; Ryan and Ketcham 2001). Thus, the pattern observed by Berles et al. (2024) may reflect functional adaptation to leaping distance, as longer leaps involve greater take‐off and landing forces (Demes et al. 1999; Nauwelaerts and Aerts 2006).

Several biases affect the study of trabecular bone. These include system‐dependent biases, such as dependence on the specific bone examined—for instance, femoral trabeculae tend to be more locomotor‐informative (Ryan and Shaw 2012; Alfieri et al. 2026). Biases may also arise from the choice of taxonomic group; significant findings come predominantly from hominid studies (e.g., Arias‐Martorell et al. 2021; Tsegai et al. 2013). Methodological biases have been identified as well. Using regularly shaped Volumes of Interest (VOIs) of trabecular bone may introduce biases related to VOI size, shape, and location (Fajardo and Müller 2001; Kivell et al. 2011; Lazenby et al. 2011). Parameter selection may further bias signal detection. Berles et al. (2024) relied on a VOI‐based approach (maximum‐size spheres, centered in the epiphysis, as detailed in Alfieri et al. 2022) and “traditional parameters”, such as DA and BV/TV, which may have obscured stronger functional signals. Beyond approaches based on VOIs and traditional parameters, alternative ones are gaining in popularity, including whole‐epiphysis analyses (Gross et al. 2014; Bachmann et al. 2022; Veneziano et al. 2021) and topological indices to capture properties measured on topological skeletons (Alfieri, Demuth, et al. 2025; Alfieri, Veneziano, et al. 2025; Veneziano et al. 2021).

**FIGURE 1:**
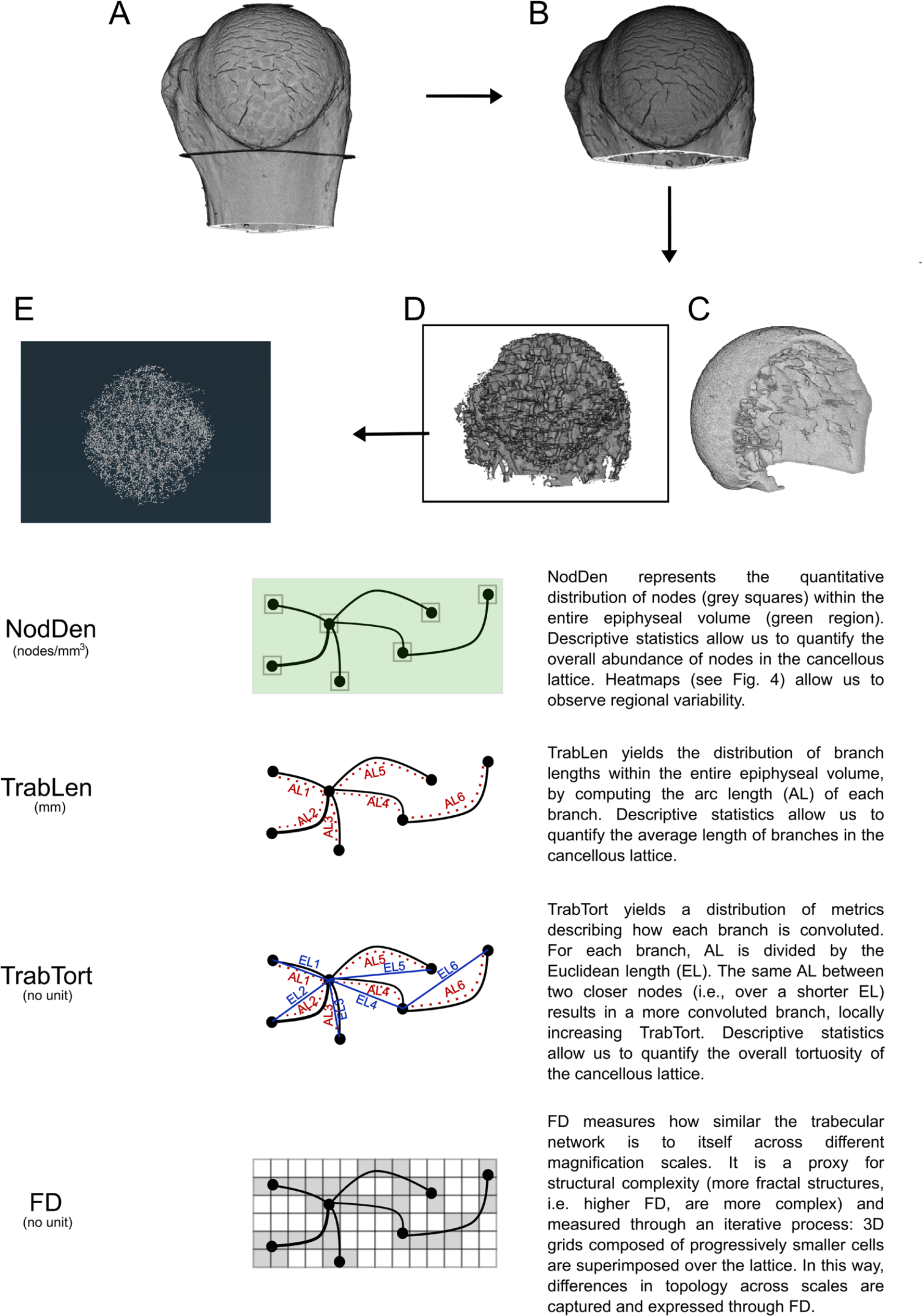
The steps of trabecular bone isolation are summarized for the proximal humeral epiphyses of *S. mystax* AMNH 188173 (the other epiphyses are shown in Figure S2). Using fixed anatomical markers (A), we cropped the epiphyses (B), isolated the ROIs (D) through the exclusion of cortical bone (C), and transformed them into topological skeletons (E). On the latter, we computed four topological properties, the geometric meanings of which are represented and explained in the figure.

Topological skeletons preserve trabecular node‐branch relationships (Alfieri, Demuth, et al. 2025; Alfieri, Veneziano, et al. 2025; Veneziano et al. 2021), quantifying complementary geometric properties of the trabecular lattice. Although their biomechanical relevance still lacks direct experimental validation, the geometric features they capture (Figure 1) are conceptually linked to mechanical loading. Node density (NodDen) reflects the spatial concentration of trabecular connections (represented by nodes in topological skeletons) within the epiphysis and conceptually parallels BV/TV; since it correlates with compressive strength, increased loading magnitude is expected to raise NodDen (Cendre et al. 1999; Veneziano et al. 2021). Trabecular length (TrabLen) should inversely relate to strength, as networks denser in nodes tend to consist of shorter trabeculae (Parkinson et al. 2012). Trabecular tortuosity (TrabTort), quantifying trabecular curvature, is hypothesized to increase elasticity and thus positively correlate with loading magnitude (Fyhrie and Zauel 2015; Roque et al. 2012; Roque and Alberich‐Bayarri 2015). Fractal dimension (FD) reflects how the trabecular network tends to preserve the same structural complexity at different degrees of magnification. High FD has been associated with low bone mineral density and mechanical inactivity; thus, FD is expected to inversely correlate with bone mineral density and, ultimately, with loading magnitude (Feder 1988; Pornprasertsuk et al. 2001; Ammann and Rizzoli 2003; Feltrin et al. 2004; Messent, Buckland‐Wright, and Blake 2005; Messent, Ward, et al. 2005).

We hypothesized that the weak trend of higher BV/TV and DA in long leapers reported by Berles et al. (2024) reflects a stronger locomotor signal attenuated by the approach based on VOIs and traditional parameters. We therefore re‐analyzed *L. nigrifrons* and *S. mystax* (long leapers) and *S. imperator* and *S. midas* (short leapers) using a whole‐epiphysis approach and quantifying both traditional parameters and topological indices. Our main aim was highlighting anatomical differences related to leaping distance. In addition, we used the composition of our sample (Table 1) to assess the effects of sex and captivity on trabecular bone, while, as justified below, the effects of other factors were considered negligible. Given the primary focus on locomotor drivers, we sampled epiphyses from forelimb and hindlimb elements, since the latter generates propulsion in take‐off (Channon et al. 2010; Demes et al. 1995, 1999), and the former absorbs landing forces (Berles et al. 2022; Garber 1991; Garber et al. 2012). We focused on the humerus and tibia, key elements in primate locomotion: the humerus bears attachment sites for numerous forelimb muscles, ligaments, and tendons (Anderson 1978), while the tibia is the primary distal leg element, involved in load bearing (Marchi 2007). The importance of the humerus and tibia is substantiated by numerous functional anatomical studies addressing them, including works on trabecular bone (e.g., Ryan and Walker 2010; Tsegai et al. 2017), or adaptations to leaping (e.g., Lad et al. 2018; Terranova 1995), due to the considerable forces expected to act on them in association with leaping dynamics (e.g., braking at landing, Lad et al. 2018).

**TABLE 1:** Specimens studied in this work, together with information on the collection they were taken from (FMNH: Field Museum of Natural History, Chicago, IL, USA; AMNH: American Museum of Natural History, New York, NY, USA), the respective catalogue number, location of provenance, sex, species to which they belong, leaping category (leaping distance), and epiphyseal elements that were included (as indicated with Y) or discarded (as indicated with N) in case of not fully fused epiphysis. Prox and dist are abbreviations for proximal and distal.

Species	Leaping category	Collection	Catalogue number	Humerus prox	Humerus dist	Tibia prox	Tibia dist	Location	Sex
*Leontocebus nigrifrons*	Long distance leaping	FMNH	122268	Y	Y	Y	Y	Zoo	m
		FMNH	122269	Y	Y	Y	Y	Zoo	f
		FMNH	122270	Y	Y	Y	Y	Zoo	f
*Saguinus mystax*	Long distance leaping	AMNH	188173	Y	Y	Y	Y	South America; Peru; Loreto; Samiria River	Unknown
		AMNH	188177	Y	Y	Y	Y	South America; Peru; Loreto; Samiria River	m
		AMNH	188178	Y	Y	Y	Y	South America; Peru; Loreto; Samiria River	f
*Saguinus imperator*	Short distance leaping	FMNH	98035	**N**	Y	Y	Y	South America, Peru, Madre de Dios, Manu, Neotropics: Altamira	f
		FMNH	98036	Y	Y	Y	Y	South America, Peru, Madre de Dios, Manu, Neotropics: Altamira	m
		FMNH	121551	Y	Y	Y	Y	Zoo	f
*Saguinus midas*	Short distance leaping	FMNH	93239	Y	Y	**N**	**N**	South America, Suriname, Nickerie, Neotropics: Kayser Gebergte Airstrip, E of Zuid R	m
		FMNH	93236	**N**	Y	**N**	**N**	South America, Suriname, Nickerie, Neotropics: Kayser Gebergte Airstrip, E of Zuid R	f
		FMNH	93516	Y	Y	Y	Y	South America, Suriname, Nickerie, Neotropics: Kayser Gebergte Airstrip, E of Zuid R	f

The aforementioned hypothesized relationships between topological indices and biomechanical loading allow us to posit how these traits should vary with different leaping patterns. Namely, if we consider the positive covariation between leaping distance and compressive limb forces (Demes et al. 1999; Nauwelaerts and Aerts 2006), we predicted that the humeri and tibiae of long leapers would exhibit higher NodDen and TrabTort, and lower TrabLen and FD, compared to short leapers. Consistent with Berles et al. (2024), we also expected long leapers to show higher BV/TV and DA than short leapers. Body mass and phylogenetic effects are assumed to be minimal because species have comparable masses (≈400–600 g; Garber and Teaford 1986) and leaping categories do not align with phylogenetic groupings (Figure S1).

## 2 Materials and Methods

### 2.1 Sample and Virtual Data Acquisition

We analyzed the humerus and tibia of 12 tamarin individuals (the same studied in Berles et al. 2024), housed at the American Museum of Natural History (AMNH, New York) and the Field Museum of Natural History (FMNH, Chicago, USA). The sample represents *Leontocebus nigrifrons* , *Saguinus midas* , *S. imperator* , and *S. mystax* (three individuals per species), allowing us to compare long and short leapers. Sex and captivity status were also considered to assess their effects on trabecular traits. Sex was known for 11 individuals (four males, seven females), with one specimen ( *S. mystax* AMNH 188173) lacking sex data. Captivity status was known for all individuals (four captive, eight wild from three South American regions; Table 1).

Bones were scanned using X‐ray micro‐computed tomography (μCT) on a Nikon Metrology XT H 225 ST (voxel size 0.0165–0.018 mm, 100 kV, 165 μA, 0.2 mm Cu filter) at SMiF (Duke University, NC), and on a GE phoenix v|tome|x s (voxel size 0.0154 mm, 170–172 kV, 79–85 μA, no filter) at UChicago PaleoCT (University of Chicago, IL, USA) (Table S1). All scans are available on MorphoSource (Boyer et al. 2016, https://www.morphosource.org). Based on incomplete internal epiphyseal fusion, three individuals were identified as likely subadults. The corresponding epiphyses were excluded from analyses, while fully fused epiphyses from the same individuals were retained (Table 1).

### 2.2 Trabecular Bone Isolation

Using VG Studio Max 3.3 (Volume Graphics, Heidelberg, Germany), humeri and tibiae were aligned into a standardized orientation following a protocol largely based on Ruff (2002) and detailed in Notes S1–S2. Epiphyses were cropped on the oriented bones by using the same anatomical markers across specimens (Figure 1A,B). For the proximal humerus, cropping extended from the most proximal point of the head to the most distal level of the anatomical neck; for the distal humerus, from the most proximal point of the capitulum to the most distal end of the bone. For the proximal tibia, cropping extended from the most proximal point of the bone to the most distal point of the lateral condyle, while for the distal tibia, it extended from the first appearance of the medial malleolus (detected going proximodistally along the image stack) to the most distal end of the bone (Figure S2).

Trabecular bone was isolated following Veneziano et al. (2021). Image stacks were binarized in ImageJ (Schneider et al. 2012, ‘Threshold’ tool), with thresholds manually adjusted after comparison with original scans to minimize misclassification. We automatically isolated trabecular bone using the ‘indianaBones’ R (v4.3.2; R Core Team 2023) package (https://github.com/AlessioVeneziano/IndianaBones) to create the trabecular regions of interest (ROI) from the whole epiphyses (Figure 1C,D). The isolation algorithm was iterated until visual comparison to stacks including cortical bone too, confirmed optimal separation. ROIs were skeletonized in Amira 6.0.0 (‘Auto Skeleton’ tool), reducing the trabecular network to topological skeletons (Figure 1E; Figure S2).

### 2.3 Quantification of Trabecular Traits

In R, we computed four topological properties for each skeleton (following Alfieri, Veneziano, et al. 2025, Veneziano et al. 2021; Note S1): NodDen (nodes/mm^3^), TrabLen (mm), TrabTort and FD (unitless). These metrics describe geometric properties of the trabecular lattice and were summarized using descriptive statistics: mean and median for NodDen and TrabTort (to account for outlier‐driven variability at the ROI margins), mean for TrabLen, while a single value already represents FD (Figure 1). Accordingly, the variables extracted were NodDen_Mean_, NodDen_Med_, TrabLen_Mean_, TrabTort_Mean_, TrabTort_Med_, and FD. Traditional trabecular parameters—BV/TV and DA—were quantified within non‐skeletonized ROIs using BoneJ routines (Doube et al. 2010, ImageJ plugin). Because BoneJ computes total volume (TV) as the full image‐stack volume rather than the ROI volume, we used the ROI volume obtained from ‘indianaBones’ isolation as TV and divided BoneJ's bone voxels count (BV) by it to calculate BV/TV.

### 2.4 Statistical Analysis

Considering the low number of species and the fact that the closely related taxa do not cluster phylogenetically by leaping distance (Figure S1), phylogenetically informed tests were not applied. Similar body mass values and comparable humerus (mean 52.94 ± 3.19 mm) and tibia lengths (mean 68.87 ± 5.27 mm; Table S1) suggested negligible allometric effects. Although the sample size (three individuals per species) limits intraspecific variation assessments, coefficients of variation (CV) were calculated (Table S4).

**FIGURE 2:**
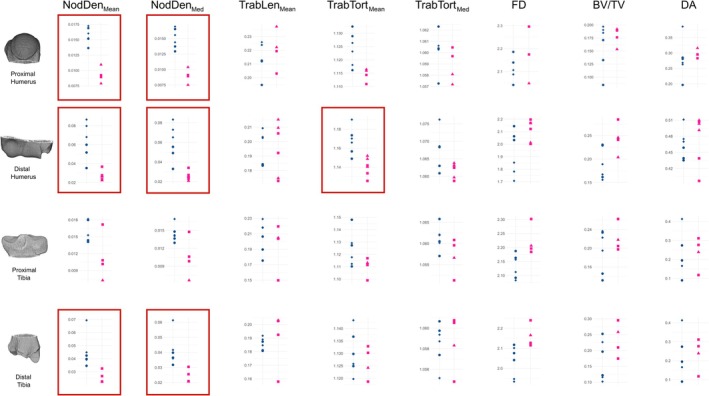
For each studied epiphysis (rows) and each trabecular trait (topological indices in columns 1–6 and traditional parameters in columns 7–8, see Figure 1 for their geometric meaning), a scatterplot showing the differences between long (blue) and short (purple) leapers is presented. Species are represented by different symbols: *L. nigrifrons* with circles, *S. mystax* with rhombi, *S. midas* with triangles, and *S. imperator* with squares. Scatterplots corresponding to traits showing significant differences between long and short leapers (based on statistical inference; see Methods) are highlighted with red rectangles.

Low sample size led us to use nonparametric Mann–Whitney *U* tests (*α* = 0.05) to compare each trabecular variable between long and short leapers (Table 2). *p* values were corrected by False Discovery Rate (FDR) to control the expected proportion of false positives deriving from multiple testing. FDR was preferred over other more conservative methods (e.g., Bonferroni) due to the preliminary nature of the study. Group differences were visualized using scatterplots (Figure 2).

**TABLE 2:** For each anatomical region studied here, that is, examined epiphyses, and for each topological index of complexity as well as traditional parameters there computed, the *p* value resulting from testing for a significant difference between long distance leaping and short distance leaping tamarins is shown. *p* values indicating significance (i.e., < 0.05) are shown in bold and highlighted with*. See Methods and above for details. The values were obtained with the Mann–Whitney *U* Test/Wilcoxon‐Rank‐Sum‐Test and after FDR correction.

Examined epiphysis	NodDen mean	NodDen median	TrabLen mean	TrabTort mean	TrabTort median	FD	BV/TV	DA
Humerus prox	0.043538*	0.043538*	0.9143	0.06096	0.391771	0.5125	0.84071	0.2886
Humerus dist	0.043538*	0.043538*	0.87274	0.043538*	0.173173	0.2286	0.075549	0.828
Tibia prox	0.2286	0.2286	0.75015	0.28867	0.2286	0.06096	0.75015	0.8407
Tibia dist	0.043538*	0.043538*	0.39177	0.750153	0.9143	0.06096	0.28867	0.75015

Abbreviations: BV/TV, bone volume fraction; DA, degree of anisotropy; dist, distal; FD, fractal dimension; NodDen, node density; prox, proximal; TrabLen, trabecular length; TrabTort, trabecular tortuosity.

**FIGURE 3:**
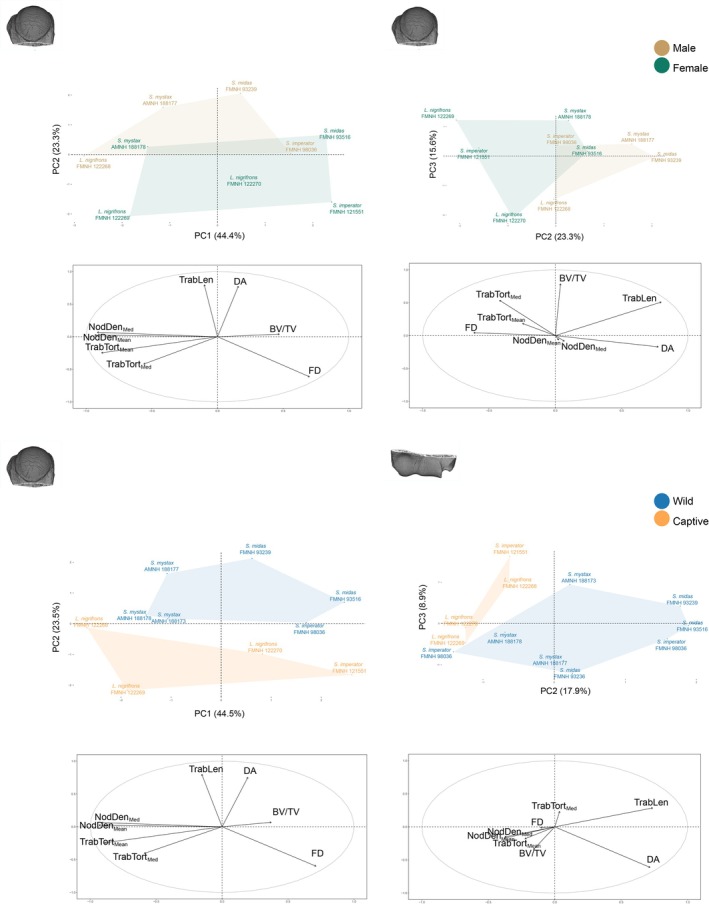
PCA biplots and the respective variable loading plots showing how and which trabecular traits are associated with sex (top) and captivity (bottom). Top left, top right, and bottom left plots refer to proximal humeral data; the bottom right plot refers to distal humeral data. All the other biplots (PC1–PC2 and PC2–PC3 for all the humeral and tibial epiphyses) are shown and described in Notes S4 and S5.

To assess potential effects of sex and captivity, Principal Component Analyses (PCA) were conducted separately for each epiphysis using all trabecular variables. Biplots of the first three PCs were generated, grouping specimens by sex and captivity status (Figure 3; Notes S4 and S5).

**FIGURE 4:**
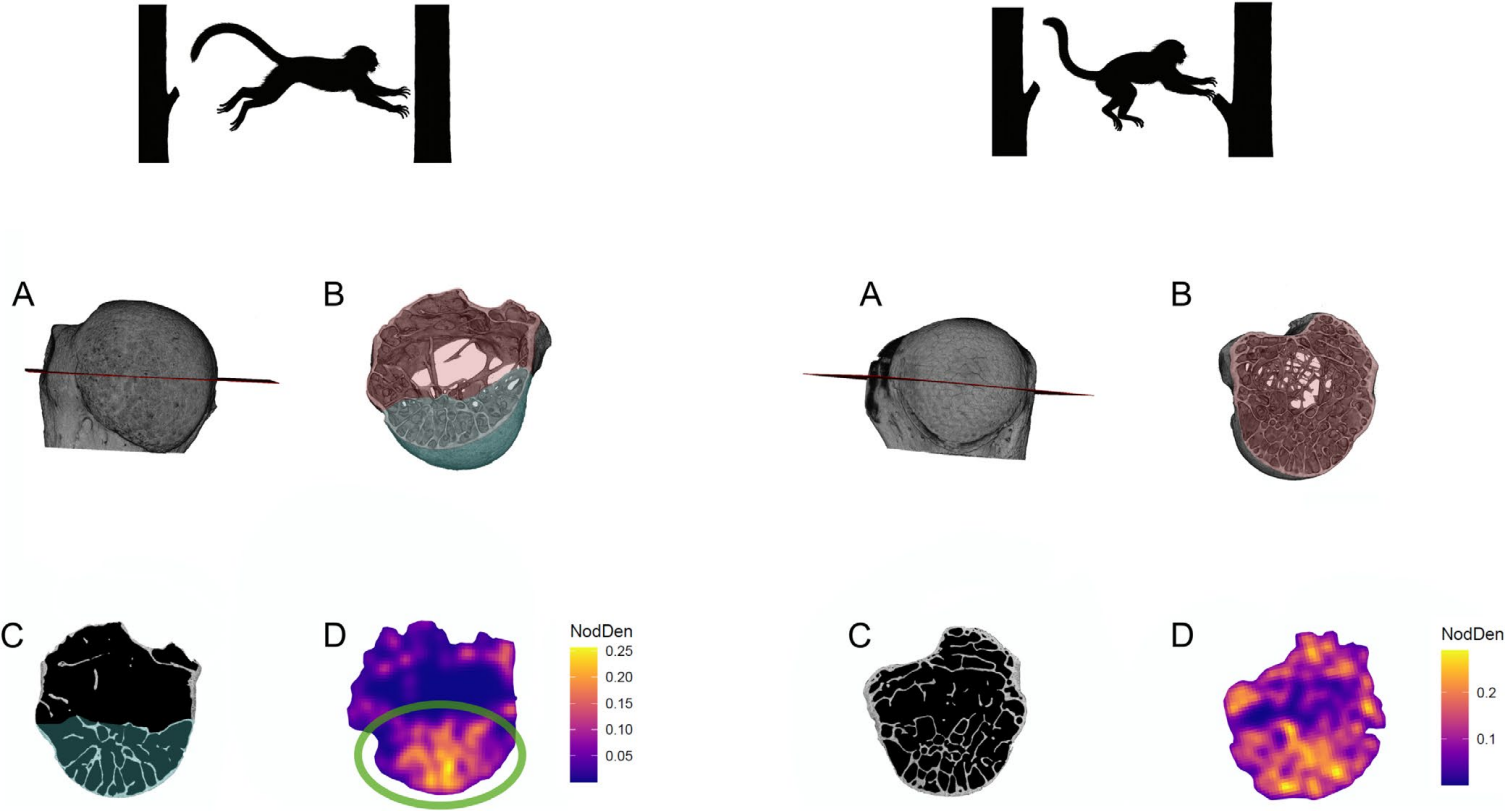
From the humeral head of a long (left, *L. nigrifrons* FMNH 122268) and a short leaper (right, *S. imperator* FMNH 98036), the trabecular structure is exposed by virtually dissecting the specimen (through the red surface in A and B) at the 50% level of the proximodistal length of the epiphysis (see Methods for the anatomical delimitation of the epiphyses). From this level, a 2D section is extracted (shown in grayscale in C). From the heatmaps generated from 3D topological skeletons, the corresponding NodDen 2D maps at the 50% level are shown in D. These maps highlight the concentration of high NodDen values frequently observed in long leapers beneath the humeral head articulation with glenoid cavity (region highlighted in light blue), in contrast to short leapers, which display more uniform NodDen values (see also Notes S6–S9).

Spatial variation in NodDen was explored using heatmaps representing its distribution across epiphyses. 2D maps were generated at 10% intervals along the proximodistal length of each epiphysis (Notes S6–S9), enabling identification of potential regional differences between long and short leapers (Figure 4).

## 3 Results

Significant correlations between leaping distance and trabecular traits are observed across both bones and in nearly all epiphyses. In particular, in three of the four epiphyses (i.e., except for the proximal tibia), long leapers exhibit trabeculae that are characterized by a significantly higher density of nodes, as indicated by increased mean and median NodDen values. Moreover, the distal humerus of long leapers shows significantly more tortuous trabeculae, reflected in higher mean TrabTort values. By contrast, no other trabecular parameters show a significant relationship with leaping distance (Table 2; Figure 2).

Spatial patterns in NodDen further support the quantitative results. Heatmaps reveal that the epiphyses of long leapers frequently contain localized regions with high node density. These regions tend to occur beneath the articular surface with the glenoid cavity in the proximal humerus and appear more variably in the distal humerus and tibia. This pattern differs from that observed in short leapers, whose epiphyses generally display more homogeneous epiphyseal NodDen distributions (Figure 4; Notes S6–S9).

Other trabecular parameters that do not correlate with leaping distance instead appear to be associated with sex and captivity status. Qualitative inspection of the PCA biplots suggests that these factors drive much of the remaining variation. In particular, males and females, as well as captive and wild individuals, tend to be characterized by distinct trabecular configurations. Females show trabeculae that are more structurally complex (higher FD values) in the proximal and distal epiphyses of both the humerus and the tibia. In the proximal humerus, females show shorter trabeculae (lower TrabLen). In the tibia, females exhibit trabeculae that are less curved (lower TrabTort), less preferentially aligned (lower DA), and characterized by a higher bone fraction (higher BV/TV). Captive individuals tend to exhibit humeral trabeculae that are shorter (lower TrabLen), more preferentially aligned (higher DA), and more structurally complex (higher FD). In the tibia, trabeculae of captive individuals are typically more curved (higher TrabTort) and characterized by higher bone fraction (higher BV/TV). Node density does not show any consistent pattern related to sex or captivity. The only possible exception is NodDen in the distal tibia, where a weak or isolated effect may be present (Notes S4 and S5).

Across all trabecular traits the coefficient of variation (CV) ranges from 0.00017 to 0.523 (mean = 0.099). When considering only traits associated with leaping distance, CV values range from 0.002 to 0.31 (mean = 0.141).

## 4 Discussion

### 4.1 Covariation of Trabecular Node Density and Tortuosity With Leaping Distance

Node density appears to be the trabecular feature showing the strongest relationship with leaping distance. Long leapers ( *L. nigrifrons* and *S. mystax* ) exhibit significantly higher NodDen (mean and median) than short leapers ( *S. midas* and *S. imperator* ) in all epiphyses except the proximal tibia (Figure 2, Table 2). NodDen is expected to be conceptually parallel to bone volume fraction (BV/TV; Tsegai et al. 2018; Note S1, but see below), which is expected to increase with higher joint load magnitude (e.g., Arias‐Martorell et al. 2021). Similar density‐related measures are also positively associated with maximum compressive strength (Cendre et al. 1999). Accordingly, higher NodDen in the epiphyses of long leapers is consistent with our functional expectations and might be a way in which tamarin trabecular structure adapts to higher loading magnitude associated with longer leaps (Demes et al. 1999).

Frequent localized regions of high node density, visible in heatmaps (Figure 4, Notes S6–S9), likely drive the overall higher NodDen in long leapers. In contrast, short leapers display more spatially homogeneous NodDen distributions. Functional relevance for this pattern is supported by the humeral head NodDen distribution, where high values consistently occur near the articular surface with the scapular glenoid cavity (Figure 4). The glenohumeral joint plays a major role in transmitting and dissipating forelimb loads in primates (Arias‐Martorell et al. 2015; Arias‐Martorell 2019; Preuschoft et al. 2010). For other epiphyses, regions of elevated NodDen are less consistently localized and therefore harder to interpret. Detailed kinematic data for the studied taxa would be required to formulate expectations of higher node density in specific regions of the epiphyses. Nonetheless, we can hypothesize that higher load magnitude associated with longer leaping distances are transmitted across epiphyses, resulting in localized increases in NodDen.

Higher trabecular tortuosity (indicated by TrabTort_Mean_) in the distal humerus of long leapers (Figure 2, Table 2) is consistent with our functional expectations. Trabecular curvature, for which TrabTort is a proxy, is inversely related to stiffness (Roque et al. 2012; Roque and Alberich‐Bayarri 2015) and more tortuous networks may enhance elasticity under higher loading magnitudes. However, this result should be interpreted cautiously, since it is restricted to a single epiphysis and is based only on mean values. Moreover, it lacks spatial visualization analogous to NodDen heatmaps due to the absence of TrabTort heatmaps. Consequently, it remains unclear whether increased tortuosity characterizes the entire distal humerus or only specific regions.

### 4.2 Other Factors Affecting Tamarin Trabecular Structure

The absence of significant relationships between leaping distance and TrabLen, FD, BV/TV, and DA (Figure 2, Table 2) may reflect the influence of sex and captivity, both previously shown to affect trabecular structure (Eckstein et al. 2007; Zack et al. 2022). Most of the patterns potentially associated with sex and captivity do not involve variables for which we hypothesized biomechanical significance (Figure 3, Notes S4 and S5). One possible exception concerns distal tibial NodDen, which apparently contributes to sex‐driven separation (Note S4). However, this pattern is driven by exceptionally high NodDen of a single specimen ( *S. mystax* AMNH 188177, belonging to a male individual) (see NodDen variable loading plots and PCA biplots; Note S4). Because this individual is neither subadult nor captive, age‐ and captivity‐related explanations can be excluded. Since *S. mystax* is a long leaper, its high NodDen is instead consistent with increased loading magnitude. Potential effects of sex and captivity were also detected for TrabTort (Notes S4 and S5). Although this occurs in the tibia—whereas we identified a relationship between TrabTort and leaping distance in the distal humerus—it represents an additional factor leading us to temper our functional interpretation of TrabTort.

The predominance of variables associated with sex and captivity over those related to leaping distance may suggest that non‐biomechanical factors account for much of trabecular variation in the studied taxa, with locomotion contributing only a secondary signal. This pattern aligns with previous studies of trabecular structure (Alfieri, Demuth, et al. 2025; Alfieri, Veneziano, et al. 2025), which similarly reported dominant non‐functional influences (e.g., phylogeny or allometry) alongside weaker locomotor signals, remarkably concerning NodDen (Alfieri, Demuth, et al. 2025) and TrabTort (Alfieri, Veneziano, et al. 2025). Alternatively, the effect of leaping distance may be substantial but difficult to detect without more refined methodologies (see also Discussion below). These could include the use of heatmaps for variables beyond NodDen, as well as other statistical tools allowing the study of whole epiphyseal variability rather than focusing only on means and medians, and larger sample sizes. The importance of the latter is exemplified by the case discussed above, in which a single outlier appears to drive sex differences.

The absence of a relationship between leaping distance and both DA and BV/TV is striking, considering the trends possibly related to leaping distance previously observed for these variables (Berles et al. 2024). DA measures a local property, that is, the degree of preferential alignment (Harrigan and Mann 1984). Hence, over whole epiphyses, the signal of DA may be less clear. Notably, the algorithm used by BoneJ to quantify DA—mean intercept length (MIL; Harrigan and Mann 1984; Odgaard 1997)—and that we used in this work, may be biased when the analyzed volumes are not fully occupied by trabecular bone. MIL estimates DA by measuring how often virtual lines intersect trabeculae in different directions; if the volume includes empty outer spaces along specific directions, these lines yield fewer intersections and artificially long intercept lengths. Consequently, directional differences in empty space may bias DA. We minimized this bias by cropping the volumes so that empty regions were equally distributed around the ROIs, ensuring that MIL‐related bias acted uniformly in all directions, therefore affecting absolute DA values but not relative differences. Nevertheless, it remains possible that this factor contributed to masking the pattern reported by Berles et al. (2024). The absence of a relationship between BV/TV and leaping distance is more puzzling to interpret, especially considering that BV/TV is expected to be conceptually related to NodDen (i.e., the parameter yielding the strongest signal related to leaping distance). Remarkably, although the two parameters measure similar properties, they are also computationally different since NodDen is computed on topological skeletons. This discrepancy may emphasize the need for combined utilization of both these parameters in comparative analyses of trabecular bone. Moreover, these unexpected differences between results for BV/TV and NodDen highlight the need for dedicated work to better understand how traditional parameters and topological indices co‐vary, and the extent to which they can be functionally paralleled.

### 4.3 Limitations and Future Directions

The small sample size and low representation of individuals within a species represents a limitation of this work. It weakened the statistical power of interspecific comparisons, allowing us to highlight only preliminary patterns that further dedicated studies need to confirm. Moreover, this issue limits our possibility to estimate intraspecific variation. For instance, tibial patterns for *S. midas* should be interpreted with caution, since the species is represented by a single specimen (due to exclusion of tibiae with unfused epiphyses). We were however able to estimate intraspecific variability by computing CV for each species and trait: in general, a CV reaching 0.46 suggests a substantial intraspecific variability (although the average value indicates an overall moderate effect, i.e., 0.096). Noticeably, if only the traits significantly related to leaping distance are considered, the maximum CV decreases to 0.31, with an average value again suggesting moderate intraspecific variability for the functional relationships that we discuss. Another potential bias may derive from the automatic trabecular bone isolation procedure that may involve unwanted inclusion of cortical bone in the studied network. It, in turn, may affect some variables (e.g., node density can be artificially increased if cortical bone is mistakenly included). However, we carefully assessed the results of each automatic trabecular isolation, checking for the potential presence of cortical bone, and we can reasonably exclude that substantial regions of cortical bone were included in the studied ROIs (see also an example of excluded cortical fraction in Figure 1, Figure S2).

Our outcomes suggest that employing a whole‐epiphysis approach and computing topological indices may help with the identification of patterns potentially reflecting fine‐grained locomotor differences, that is, different leaping distances. By using this alternative approach, we highlighted trends that Berles et al. (2024), who examined the same specimens using VOIs and traditional parameters, did not find. On the other hand, our approach did not identify stronger signals for the preliminary patterns reported by Berles et al. We therefore propose that future morpho‐functional studies of trabecular bone should combine both traditional and recent methodologies, as the two can be complementary and provide a more complete framework. It should also be noted that, despite the potential improvements offered by whole‐epiphysis techniques, several advantages remain associated with VOI approaches. For instance, VOIs offer easier reproducibility, lower computational demands, and better representation of regional properties (e.g., DA) (Kivell et al. 2011).

## 5 Conclusions

On whole humeral and tibial epiphyses of tamarins, we quantified traditional parameters and topological indices to assess relationships between trabecular structure, leaping distance, and non‐locomotor factors (sex and captivity). Node density (NodDen) and trabecular tortuosity (TrabTort) followed our functional expectations, with long distance leapers ( *L. nigrifrons* , *S. mystax* ) showing higher values than short distance leapers ( *S. midas* , *S. imperator* ). However, the TrabTort signal was limited to a single epiphysis and lacked support from intra‐epiphyseal heatmaps, whereas NodDen showed consistent high‐value regions, mainly in the humeral head, suggesting potential functional relevance. These putative locomotion‐related effects were secondary to stronger patterns associated with non‐biomechanical factors: trabecular length, fractal dimension, bone volume fraction, and degree of anisotropy were more strongly associated with sex and captivity. Thus, leaping distance possibly influences specific trabecular features—mostly NodDen—in tamarins, but the overall trabecular configuration is likely driven by non‐functional factors. While whole‐epiphysis analyses and topological indices provide a powerful framework for characterizing trabecular structure, traditional VOI‐based approaches and traditional metrics remain valuable, as they reveal complementary patterns.

## Author Contributions

**Uyen Nguyen:** conceptualization (supporting), data curation (lead), formal analysis (lead), funding acquisition (supporting), investigation (lead), methodology (supporting), project administration (supporting), resources (supporting), software (lead), supervision (supporting), validation (supporting), visualization (lead), writing – original draft (lead), writing – review and editing (equal). **Fabio Alfieri:** conceptualization (lead), data curation (lead), formal analysis (lead), funding acquisition (supporting), investigation (lead), methodology (lead), project administration (lead), resources (lead), software (lead), supervision (lead), validation (lead), visualization (lead), writing – original draft (lead), writing – review and editing (equal). **Alessio Veneziano:** formal analysis (equal), investigation (equal), methodology (equal), software (equal), visualization (equal). **Annika Licht:** conceptualization (supporting), data curation (lead), formal analysis (lead), funding acquisition (supporting), investigation (lead), methodology (supporting), project administration (supporting), resources (supporting), software (lead), supervision (supporting), validation (supporting), visualization (supporting). **John A. Nyakatura:** conceptualization (lead), data curation (supporting), formal analysis (supporting), funding acquisition (lead), investigation (supporting), methodology (supporting), project administration (lead), resources (lead), software (supporting), supervision (lead), validation (supporting), visualization (supporting), writing – original draft (supporting), writing – review and editing (equal).

## Funding

This work was funded by Deutsche Forschungsgemeinschaft, NY 63/2‐1; Fabio Alfieri was financially supported by Schweizerischer Nationalfonds zur Förderung der Wissenschaftlichen Forschung, project grant TMPFP3_217022.

## Ethics Statement

No human remains are involved in this work. We studied virtual data derived from animal remains represented by bones, which are stored and collected as dried specimens in museum collections (as detailed in the Methods section). These bones were sampled and CT‐scanned for previous analyses (Berles et al. 2024) and we used these already generated virtual data to perform this analysis. Hence, our procedures to study animal remains did not require any ethical review.

## Conflicts of Interest

The authors declare no conflicts of interest.

## Supporting information


**Figure S1:** Topology of the phylogenetic relationships among the four examined tamarin species.
**Table S1:** Bone lengths and scanning information of the 12 analyzed humeri/tibiae.
**Figure S2:** Trabecular bone isolation is summarized for the humeral (A. proximal; B. distal) and the tibial (C. proximal; D. distal) epiphyses
**Table S2:** Raw results for the topological indices and traditional trabecular variables extracted from humeral epiphyses.
**Table S3:** Raw results for the topological indices and traditional trabecular variables extracted from tibial epiphyses.
**Table S4:** At the four studied epiphyses, for each variable (both topological indices and traditional parameters) and for the specimens representing each species, mean, standard deviation and coefficient of variation are shown.

## Data Availability

Supporting Information is downloadable from Figshare (https://doi.org/10.6084/m9.figshare.27332193) (Nguyen et al. 2025). The R code available for Alfieri, Veneziano, et al. 2025 (downloadable from Figshare at https://doi.org/10.6084/m9.figshare.24147840) enables the extraction of the topological indices using “indianaBones”. The μCT scans of the humeri and the tibiae studied in this work are freely downloadable from MorphoSource (https://www.morphosource.org/). The R package “indianaBones” is available at https://github.com/AlessioVeneziano/IndianaBones and at https://zenodo.org/record/7615165.
