# Supplementary material for: Whole‐Epiphysis Trabecular Bone in Tamarin Limbs Suggests Effects of Leaping Distance Alongside Non‐Biomechanical Factors
Source: Am J Biol Anthropol. 2026 Jun 8;190(2):e70293. doi: 10.1002/ajpa.70293 (PMC13246452; doi:10.1002/ajpa.70293)
Supplement: Supplementary file 1 — Figure S1: Topology of the phylogenetic relationships among the four examined tamarin species. Table S1: Bone lengths and scanning information of the 12 analyzed humeri/tibiae. Figure S2: Trabecular bone isolation is summarized for the humeral (A. proximal; B. distal) and the tibial (C. proximal; D. distal) epiphyses Table S2: Raw results for the topological indices and traditional trabecular variables extracted from humeral epiphyses. Table S3: Raw results for the topological indices and traditional trabecular variables extracted from tibial epiphyses. Table S4: At the four studied epiphyses, for each variable (both topological indices and traditional parameters) and for the specimens representing each species, mean, standard deviation and coefficient of variation are shown. [file AJPA-190-e70293-s001.pdf]

Whole-epiphysis trabecular bone in tamarin limbs suggests effects of leaping distance alongside non-biomechanical factors

Uyen Nguyen[1,+,*], Fabio Alfieri[2,3,1,4,*], Alessio Veneziano[5], Annika Licht[1], John A. Nyakatura[1]

[1]: Comparative Zoology, Institute of Biology, Humboldt University, Philippstraße 13, 10115 Berlin, Germany

[2]: Institute of Ecology and Evolution, Universität Bern, Bern, Switzerland

[3]: Department of Earth Sciences, University of Cambridge, Cambridge, UK

[4]: Museum Für Naturkunde, Leibniz-Institut Für Evolutions- Und Biodiversitätsforschung, Berlin, Germany

[5]: Archéozoologie, Archéobotanique: Sociétés, Pratiques et Environnements (AASPE), Muséum National d'Histoire Naturelle, CNRS, Paris, France

[+]: u.nguyen@izw-berlin.de

[*]: These authors contributed equally.

Supplementary Material

Note S1: As further detailed in Veneziano et al. 2021 and Alfieri et al. 2025, the four topological indices quantified on whole epiphyses inform on structural properties that, in turn, have recognised biomechanical significance.

1. **Node density**: this index derives from trabecular density, which is itself a conceptual generalisation of BV/TV. For instance, Tsegai et al. (2018) computed BV/TV by exploiting a grid encompassing the bone and then using interpolation to extract a continuous BV/TV map. If TV is replaced with a unit volume, the same procedure results in a density map. If, in addition, a skeletonisation procedure is applied (see Methods in the main text), the resulting density distribution includes only topological properties, yielding a node density distribution. Node density informs on the distribution of the number of nodes within a given volume (Veneziano et al. 2021) and was computed by estimating a kernel density over a 3D grid corresponding to the whole epiphysis in this study (TV). Hence, since node density is indirectly related to BV/TV, we can assume that they respond similarly to biomechanical stimuli - namely, that both are positively related to increased loading - as suggested by preliminary results on the femoral head of humans, African apes, quadrupeds, and brachiators (Veneziano et al. 2021). Further supporting this positive relationship between node density and increased loading, Cendre et al. (1999) found that node density per total volume increases in association with higher maximum compressive strength.
2. **Trabecular length**: following skeletonisation, trabecular length can be computed as the geodesic length of a branch delimited by a pair of nodes. The potential functional significance of trabecular length lies in its expected negative correlation with node density; that is, denser structures are generally characterised by shorter trabeculae. Moreover, trabecular length itself may be related to bone strength (Parkinson et al. 2012). We therefore assume that shorter trabeculae are expected in association with denser structures in response to increased loading.
3. **Trabecular tortuosity**: the computation of trabecular length is required to derive trabecular tortuosity, which serves as a proxy for the degree to which trabeculae are curved and convoluted. Trabecular tortuosity is obtained by dividing the trabecular length between two nodes (as

computed above) by the Euclidean distance between the same two nodes. In other words, if a given arc length occurs over a shorter Euclidean distance, the branch (and thus the trabecula) is more sinuous, contributing to a more tortuous overall structure. Tortuosity has been shown to capture relevant mechanical features (Fyhrie and Zauel 2015). Specifically, tortuosity affects how flexibly trabeculae respond to mechanical loads: higher tortuosity correlates with lower stiffness, that is, higher elasticity (Roque et al. 2012; Roque and Alberich-Bayarri 2015). Decreased stiffness, coupled with increased tortuosity and flexibility, suggests a more elastic structure, which may represent a response to higher loading. Hence, tortuosity may positively correlate with loading.

4. **Fractal dimension**: it measures structural complexity, specifically the extent to which a structure remains similar to itself across different degrees of magnification. The more a structure resembles itself at varying magnifications, the closer it approaches an ideal fractal network (Feder 1988; Pornprasertsuk et al. 2001). This concept has been applied to trabecular architecture to investigate mechanical aspects related to fracture risk and osseous pathologies in humans (Feltrin et al. 2004; Messent et al. 2005b,a), as well as bone density, which informs on bone strength (Ammann and Rizzoli 2003). FD relates to density, and here we assume a negative correlation between the two based on experimental evidence: when bone is experimentally subjected to periods of inactivity, it responds by decreasing density and increasing FD (Pornprasertsuk et al. 2001). Accordingly, we expect FD to decrease in association with increased loading.

Bibliography:

Alfieri, F., A. Veneziano, D. Panetta, P. A. Salvadori, E. Amson, and D. Marchi. 2025. The relationship between primate distal fibula trabecular architecture and arboreality, phylogeny and size. Journal of Anatomy 00:1–29.

Ammann, P., and R. Rizzoli. 2003. Bone strength and its determinants. Osteoporosis International 14:13–18.

Cendre, E., D. Mitton, J. P. Roux, M. E. Arlot, F. Duboeuf, B. Burt-Pichat, C. Rumelhart, G. Peix, and P. J. Meunier. 1999. High-resolution computed tomography for architectural characterization of human lumbar cancellous bone: relationships with histomorphometry and biomechanics. Osteoporosis International 10:353–360.

Feder, J. 1988. Fractals. New York: Plenum.

Feltrin, G. P., R. Stramare, D. Miotto, D. Giacomini, and C. Saccavini. 2004. Bone fractal analysis. Current Osteoporosis Reports 2(2):53–58.

Fyhrie, D. P., and R. Zauel. 2015. Directional tortuosity as a predictor of modulus damage for vertebral cancellous bone. Journal of Biomechanical Engineering 137(1):011007.

Messent, E. A., J. C. Buckland-Wright, and G. M. Blake. 2005a. Fractal analysis of trabecular bone in knee osteoarthritis (OA) is a more sensitive marker of disease status than bone mineral density (BMD). Calcified Tissue International 76:419–425.

Messent, E. A., R. J. Ward, C. J. Tonkin, and C. Buckland-Wright. 2005b. Tibial cancellous bone changes in patients with knee osteoarthritis. A short-term longitudinal study using fractal signature analysis. Osteoarthritis and Cartilage 13(6):463–470.

Parkinson, I. H., A. Badiei, M. Stauber, J. Codrington, R. Müller, and N. L. Fazzalari. 2012. Vertebral body bone strength: the contribution of individual trabecular element morphology. Osteoporosis International 23:1957–1965.

Pornprasertsuk, S., J. B. Ludlow, R. L. Webber, D. A. Tyndall, A. I. Sanhueza, and M. Yamauchi. 2001. Fractal dimension analysis of weight-bearing bones of rats during skeletal unloading. Bone 29(2):180–184.

Roque, W. L., and A. Alberich-Bayarri. 2015. Tortuosity Influence on the Trabecular Bone Elasticity and Mechanical Competence. Pp. 173–191 *in* Developments in Medical Image Processing and Computational Vision. R. N. Jorge, J. M. Tavares (Eds.), Cham: Springer.

Roque, W. L., K. Arcaro, and A. Alberich-Bayarri. 2012. Mechanical competence of bone: a new parameter to grade trabecular bone fragility from tortuosity and elasticity. IEEE Transactions on Biomedical Engineering 60(5):1363–1370.

Ruff, C. B. 2002. Long bone articular and diaphyseal structure in old world monkeys and apes. I: locomotor effects. Am. J. Phys. Anthropol. 119:305–342.

Tsegai, Z. J., M. M. Skinner, D. H. Pahr, J.-J. Hublin, and T. L. Kivell. 2018. Ontogeny and variability of trabecular bone in the chimpanzee humerus, femur and tibia. Am J Phys Anthropol 167:713–736.

Veneziano, A., M. Cazenave, F. Alfieri, D. Panetta, and D. Marchi. 2021. Novel strategies for the characterization of cancellous bone morphology: Virtual isolation and analysis. Am J Phys Anthropol ajpa.24272.

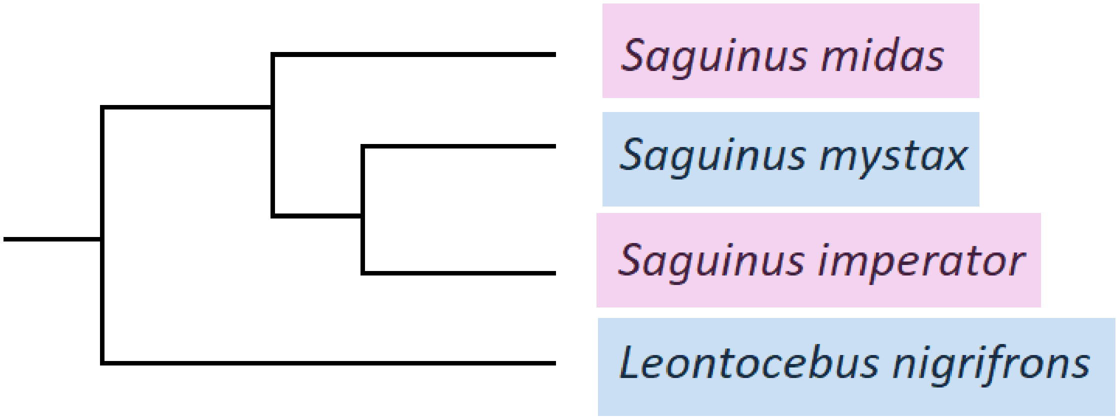

Fig. S1. Topology of the phylogenetic relationships among the four examined tamarin species. Long leapers are highlighted in blue, whereas short leapers are highlighted in purple. Because leaping-distance categories do not correspond to phylogenetic groups, phylogenetic effects can be considered distinguishable from those of leaping distance.

Table S1: Bone lengths and scanning information of the twelve analyzed humeri/tibiae

Species	Specimen	Acquired at	CT-scanning resolution (mm)	CT scanning Voltage (kV)	CT scanning current (uA)	Filter	Humerus length (mm)	Tibia length (mm)
L.nigrifrons	FMNH 122268	UChicago	0.0165	100	165	0.2mm Cu	52.338	70.422
L.nigrifrons	FMNH 122269	UChicago	0.0165	100	165	0.2mm Cu	52.420	66.478
L.nigrifrons	FMNH 122270	UChicago	0.0165	100	165	0.2mm Cu	51.348	66.709
S.mystax	AMNH 188173	SMiF	0.015487	170	79	No	49.745	63.745
S.mystax	AMNH 188177	SMiF	0.015487	172	85	No	46.895	59.641
S.mystax	AMNH 188178	SMiF	0.015487	170	83	No	50.287	64.426
S.imperator	FMNH 98035	UChicago	0.0165	100	165	0.2mm Cu	53.542	67.188
S.imperator	FMNH 98036	UChicago	0.0165	100	165	0.2mm Cu	53.592	66.165
S.imperator	FMNH 121551	UChicago	0.018	100	165	0.2mm Cu	55.836	70.884
S.midas	FMNH 93239	UChicago	0.0165	100	165	0.2mm Cu	57.139	-
S.midas	FMNH 93236	UChicago	0.0165	100	165	0.2mm Cu	55.209	-
S.midas	FMNH 93516	UChicago	0.0165	100	165	0.2mm Cu	56.925	75.438

Note S2: We oriented the humeri following Appendix Fig. 2 of Ruff C. (*Am J Phys Anth*, 119(4), 305-342. 2002). In brief, the bone was first positioned with the posterior surface (highlighted in orange in Fig. A) pointing downward. To achieve this, the bone was observed in distal view (Fig. A) and rotated in the transverse plane (defined by the x- and y-axes highlighted in green in Fig. A) until the midpoints of the lateral and medial epicondyles lay along the x-axis. In this way, the humerus was oriented in the transverse plane. To orient the humeri in the coronal plane (defined by the x- and z-axes highlighted in green in Fig. B, showing the bone in posterior view), we aligned the midpoint of the mediolateral (ML) distance along the shaft at the level of the surgical neck (shown proximally in Fig. B with two intersecting blue arrows) and the point of intersection between the trochlea and the capitulum (approximately corresponding to the point where the y-axis intersects the bone on the anterior surface in Fig. A, highlighted by a black ellipse) with the z-axis. Similarly, to orient the humeri in the sagittal plane (which is perpendicular to the coronal plane and is not shown here but, like the coronal plane, displays the bone along its longest dimension, i.e. proximodistally), we aligned the midpoint of the anteroposterior (AP) distance along the shaft at the level of the surgical neck and the midpoint of the AP distance at the level of the lateral lip of the trochlea (approximately corresponding to the purple ellipse in Fig. A) with the z-axis.

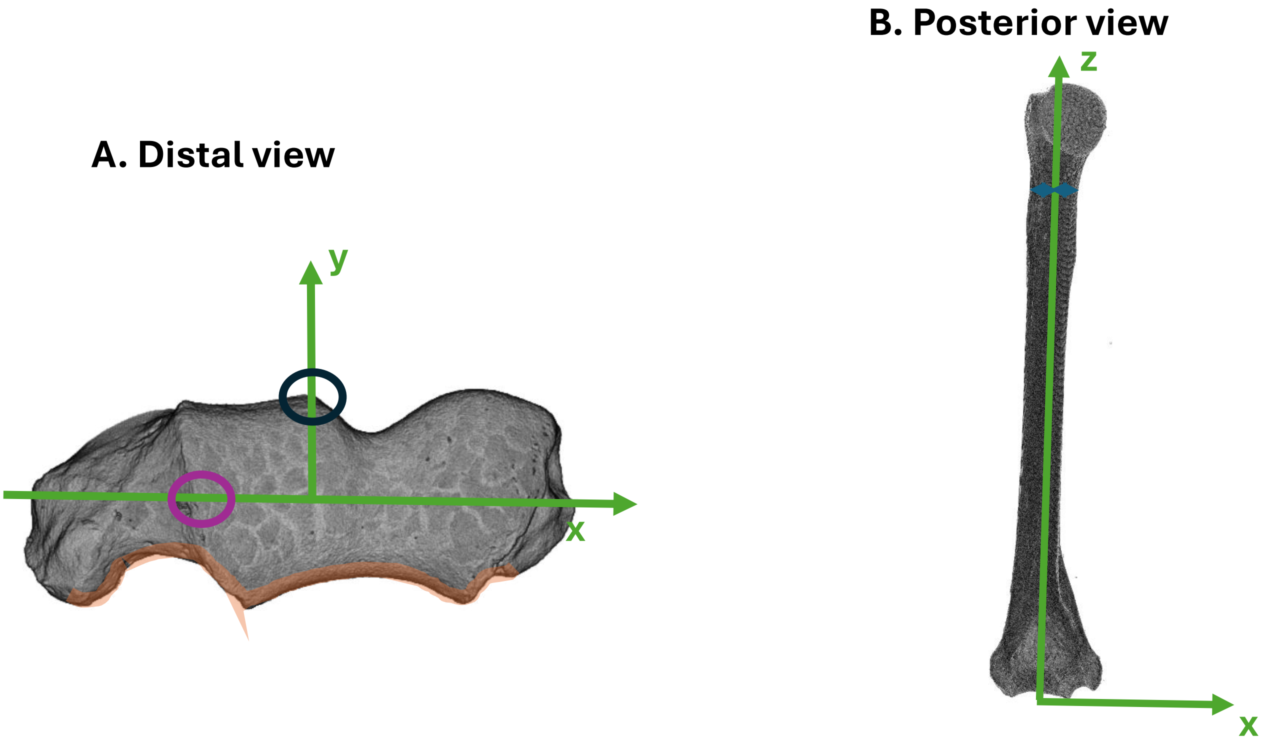

Note S3: We oriented the tibiae following Appendix Fig. 2 of Ruff C. (*Am J Phys Anth*, 119(4), 305-342. 2002). In brief, the bone was first placed with the posterior surface (highlighted in orange in Fig. A), pointing down. To do this, we observed the bone in proximal view (Fig. A) and we rotated it in the transverse plane (defined by the x- and y-axes highlighted in green in Fig. A) until the mid-points of the anteroposterior (AP) lengths of the lateral and medial condyles were at the same distance from the x axis. In Figure A, the medial and lateral condyles are highlighted in light blue, the AP lengths of the lateral and medial condyles are represented by black dashed lines, their mid-points are represented by the intersection between the black dashed lines and the horizontal yellow dashed line. In other words, orienting the tibia following these criteria, corresponds to make the AP lengths parallel to the y-axis and the yellow dashed line parallel to the x-axis. In this way, the tibia is oriented in the transverse plane. To orient the tibiae in the coronal plane (defined by the x- and z-axes highlighted in green in Fig. B, showing the bone in posterior view) we aligned the mid-points of the proximal and distal tibial plateaus (represented by the intersection of the blue arrows, both proximally and distally) with the z-axis (Fig. B). Similarly, to orient the tibiae in the sagittal plane (which is perpendicular to the coronal and is not shown here but, like the coronal plane, displays the bone along its longest size, i.e. proximodistally) we aligned the mid-point of the proximal tibial plateau (approximately corresponding to the intersection between the y-axis and the yellow dashed line in Fig. A) and the mid-point of the tibio-talar surface (shown with the intersection of the two yellow arrows in Fig. C, while the surface is highlighted in blue) with the z-axis.

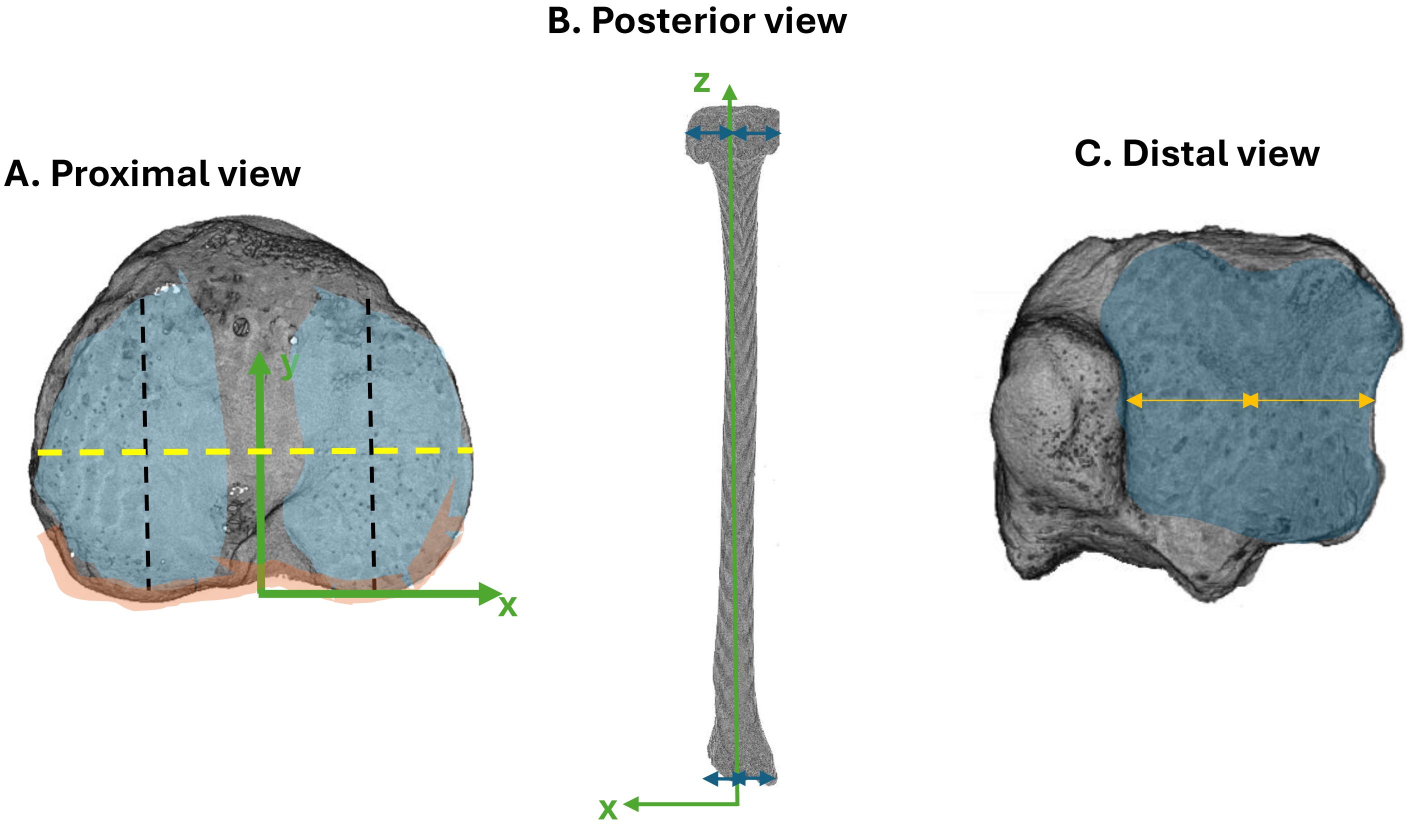

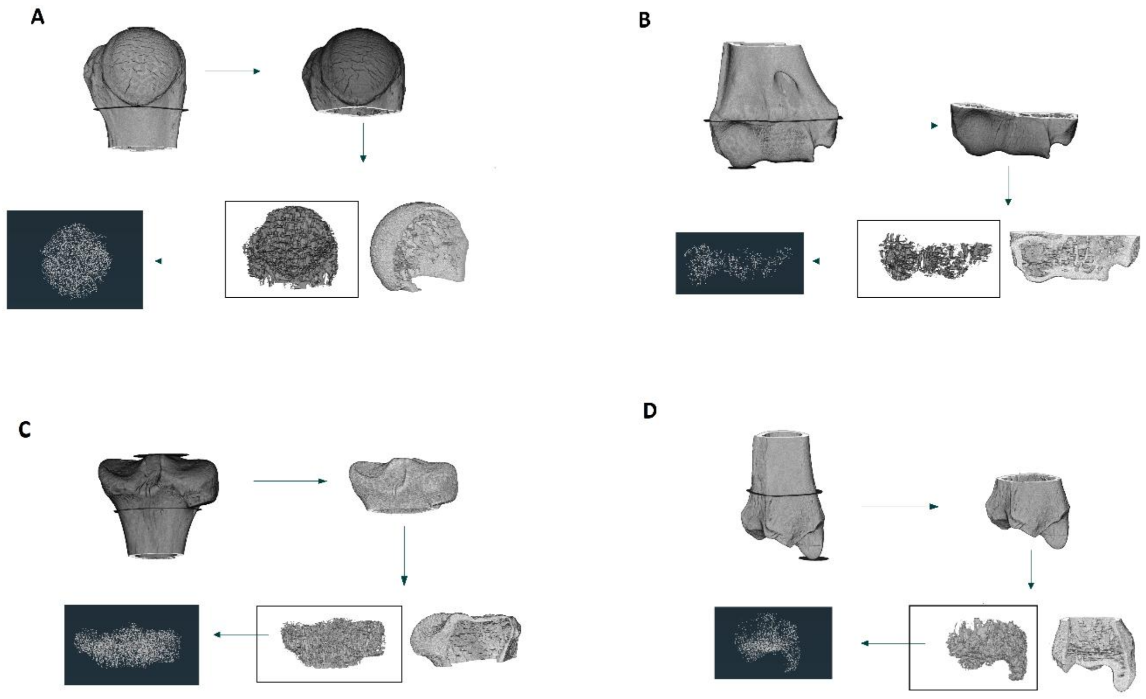

Fig. S2. Trabecular bone isolation is summarized for the humeral (A. proximal; B. distal) and the tibial (C. proximal; D. distal) whole epiphyses of *S. mystax* AMNH 188173. From fixed anatomical markers on proximal (first step in A, C) and distal epiphyses (first step in B, D), we cropped the epiphyses (second step in A-D), we isolated the ROIs (through the exclusion of cortical bone; third step in A-D) and we transformed them into topological skeletons (four step in A-D).

Table S2: Raw results for the topological indices and traditional trabecular variables extracted from humeral epiphyses

Humeral proximal epiphyses

Species	Specimen	NodDen_mean	NodDen_median	TrabLen	TrabTort_mean	TrabTort_median	FD	DA	BV/TV
L. nigrifrons	FMNH_122268	0.01689643	0.01653007	0.21250315	1.12889552	1.06037447	2.04154152	0.2852734222	0.0850497933
L. nigrifrons	FMNH_122269	0.01516662	0.01372665	0.21193715	1.13252471	1.06231243	2.13937887	0.196463575	0.1710026627
L. nigrifrons	FMNH_122270	0.01364965	0.01293283	0.19470246	1.11614431	1.05726686	2.18581409	0.2811093387	0.1326528135
S.mystax	AMNH_188173	0.01526758	0.01440248	0.22393557	1.1231462	1.06035569	2.08736926	0.26318086	0.190463774
S.mystax	AMNH_188177	0.01725113	0.01693791	0.22384094	1.11809448	1.06061091	2.10629236	0.3860114934	0.1848945299
S.mystax	AMNH_188178	0.01602279	0.01556561	0.2259206	1.12635744	1.06026407	2.08776903	0.2685756361	0.196831586
S.imperator	FMNH_98036	0.00923939	0.00911521	0.21936717	1.11434779	1.05968505	2.17469472	0.295567181	0.1762331189
S.imperator	FMNH_121551	0.00893315	0.00892911	0.20300627	1.11092799	1.06046037	2.29642146	0.283006459	0.189336165
S.midas	FMNH_93239	0.01095205	0.01038249	0.2373098	1.11644196	1.05808405	2.0485198	0.3160346500	0.1534299294
S.midas	FMNH_93516	0.00788591	0.00751383	0.22221041	1.11589279	1.05718546	2.15454963	0.294618937	0.1920903086

Humeral distal epiphyses

Species	Specimen	NodDen_mean	NodDen_median	TrabLen	TrabTort_mean	TrabTort_median	FD	DA	BV/TV
L.nigrifrons	FMNH_122268	0.0352776	0.032986	0.18374689	1.15664737	1.06300132	2.06300405	0.43524190	0.1616580373
L.nigrifrons	FMNH_122269	0.05989379	0.05543621	0.18433095	1.17358021	1.06837196	2.0327086	0.458805	0.23047478
L.nigrifrons	FMNH_122270	0.05170705	0.04914879	0.18342762	1.14890554	1.06089379	2.14386731	0.4392291	0.2290525036
S.mystax	AMNH_188173	0.0680265	0.06518007	0.2092837	1.16592152	1.07622962	1.78149961	0.4700684364	0.1672183554
S.mystax	AMNH_188177	0.07973235	0.07293333	0.20254803	1.17013673	1.06862335	1.85222329	0.5111801200	0.1889769505
S.mystax	AMNH_188178	0.08653515	0.0832564	0.20318213	1.19017183	1.06832703	1.7064093	0.4750209845	0.1558758403
S.imperator	FMNH_98035	0.03672757	0.03410344	0.1728478	1.14182806	1.05870741	2.16893836	0.438925506	0.2842036962
S.imperator	FMNH_98036	0.02766595	0.02674902	0.20570805	1.13358862	1.0624972	2.12157108	0.5068928240	0.240996279
S.imperator	FMNH_121551	0.0235465	0.02276308	0.1921667	1.12556552	1.0624795	2.19677409	0.3969649846	0.2421827378
S.midas	FMNH_93239	0.02680486	0.02575404	0.21526579	1.15195741	1.06379119	2.01214074	0.491423762	0.2038745845
S.midas	FMNH_93236	0.02232845	0.02076193	0.17472116	1.14034822	1.05969714	2.16775709	0.5033395820	0.2458056698
S.midas	FMNH_93516	0.02628588	0.02477753	0.20956465	1.14872888	1.06317054	2.00017572	0.509815033	0.203715211

Table S3: Raw results for the topological indices and traditional trabecular variables extracted from tibial epiphyses

Tibial proximal epiphyses

Species	Specimen	NodDen_mean	NodDen_median	TrabLen	TrabTort_mean	TrabTort_median	FD	DA	BV/TV
L. nigrifrons	FMNH_122268	0.01797327	0.01480379	0.17531275	1.14804527	1.06579176	2.09205693	0.3700112576	0.1313727278
L. nigrifrons	FMNH_122269	0.01409536	0.01292352	0.20635195	1.12730837	1.06052408	2.1873939	0.3735742434	0.2335995473
L. nigrifrons	FMNH_122270	0.0143671	0.01363609	0.18962899	1.1102672	1.05705751	2.16318145	0.3845664267	0.1456952133
S. mystax	AMNH_188173	0.01441196	0.01407099	0.21777114	1.11769237	1.06015572	2.15681309	0.3880705065	0.2228000773
S. mystax	AMNH_188177	0.01815416	0.01690632	0.22906937	1.11233111	1.06197188	2.0827739	0.5106507859	0.1949843215
S. mystax	AMNH_188178	0.01528028	0.01421273	0.2182397	1.12916652	1.0621106	2.11240575	0.3965903276	0.2366615044
S. imperator	FMNH_98035	0.01717169	0.0147536	0.14977164	1.09897389	1.05131301	2.30187632	0.2887047289	0.2625664815
S. imperator	FMNH_98036	0.01081666	0.01060486	0.21948656	1.11141683	1.0608389	2.18448485	0.4355622472	0.1975674889
S. imperator	FMNH_121551	0.01015415	0.00984192	0.20363324	1.11686282	1.05958448	2.19641234	0.4309485260	0.2047152717
S. midas	FMNH_93516	0.00725621	0.00664052	0.20479126	1.11314952	1.05663358	2.20665397	0.3187520116	0.2180968338

Tibial distal epiphyses

Species	Specimen	NodDen_mean	NodDen_median	TrabLen	TrabTort_mean	TrabTort_median	FD	DA	BV/TV
L. nigrifrons	FMNH_122268	0.03457818	0.03177105	0.18059852	1.12979591	1.0597556	2.07597361	0.19526977292	0.121813684
L. nigrifrons	FMNH_122269	0.03979007	0.03653906	0.18737309	1.1367426	1.06066531	2.04143224	0.274289744009	0.1964030982
L. nigrifrons	FMNH_122270	0.04239635	0.03984305	0.18101821	1.12489872	1.05739511	2.11773648	0.089978423116	0.2524142244
S. mystax	AMNH_188173	0.03922473	0.03600135	0.18469391	1.11973299	1.05514965	2.10158784	0.165989041171	0.2264232683
S. mystax	AMNH_188177	0.0693735	0.06131289	0.19172642	1.14331347	1.05936652	1.94719523	0.413337967155	0.1018531273
S. mystax	AMNH_188178	0.04486076	0.04167675	0.18895839	1.12580681	1.05872056	1.93388858	0.193374758788	0.1159032459
S. imperator	FMNH_98035	0.03250676	0.03043884	0.15793409	1.11866975	1.05480596	2.24000748	0.117912190558	0.2952585792
S. imperator	FMNH_98036	0.02248276	0.02094895	0.20258895	1.13024434	1.06076584	2.11511061	0.311663592876	0.1745774121
S. imperator	FMNH_121551	0.02670999	0.0255503	0.19245365	1.13287305	1.06052225	2.12716714	0.276714458429	0.2095902606
S. midas	FMNH_93516	0.02256071	0.02063783	0.20311824	1.12422849	1.05831849	2.16550041	0.238867282347	0.2583197171

Table S4: At the four studied epiphyses, for each variable (both topological indices and traditional parameters) and for the specimens representing each species (if there were data for at least 2 individuals) we computed the mean ($\bar{x}$), the standard deviation (SD) and the coefficient of variation (CV).

**Humerus proximal**	NodDen_mean			NodDen_median			TrabLen			TrabTort_mean			TrabTort_median			FD			DA			BV/TV		
	$\bar{x}$	SD	CV	$\bar{x}$	SD	CV	$\bar{x}$	SD	CV	$\bar{x}$	SD	CV	$\bar{x}$	SD	CV	$\bar{x}$	SD	CV	$\bar{x}$	SD	CV	$\bar{x}$	SD	CV
Leontocebus nigrifrons	0.015	0.002	0.107	0.014	0.002	0.131	0.206	0.010	0.049	1.126	0.009	0.008	1.060	0.003	0.002	2.122	0.074	0.035	0.254	0.050	0.197	0.130	0.043	0.332
Saguinus mystax	0.016	0.001	0.062	0.016	0.001	0.081	0.225	0.001	0.005	1.123	0.004	0.004	1.060	0.000	0.000	2.094	0.011	0.005	0.306	0.069	0.227	0.191	0.006	0.031
Saguinus imperator	0.009	0.000	0.024	0.009	0.000	0.015	0.211	0.012	0.055	1.113	0.002	0.002	1.060	0.001	0.001	2.236	0.086	0.039	0.289	0.009	0.031	0.183	0.009	0.051
Saguinus midas	0.009	0.002	0.230	0.009	0.002	0.227	0.230	0.011	0.046	1.116	0.000	0.000	1.058	0.001	0.001	2.102	0.075	0.036	0.305	0.015	0.050	0.173	0.027	0.158

**Humerus distal**	NodDen_mean			NodDen_median			TrabLen			TrabTort_mean			TrabTort_median			FD			DA			BV/TV		
	$\bar{x}$	SD	CV	$\bar{x}$	SD	CV	$\bar{x}$	SD	CV	$\bar{x}$	SD	CV	$\bar{x}$	SD	CV	$\bar{x}$	SD	CV	$\bar{x}$	SD	CV	$\bar{x}$	SD	CV
Leontocebus nigrifrons	0.049	0.013	0.256	0.046	0.012	0.253	0.184	0.000	0.002	1.160	0.013	0.011	1.064	0.004	0.004	2.080	0.057	0.028	0.444	0.013	0.028	0.207	0.039	0.190
Saguinus mystax	0.078	0.009	0.120	0.074	0.009	0.123	0.205	0.004	0.018	1.175	0.013	0.011	1.071	0.004	0.004	1.780	0.073	0.041	0.485	0.022	0.046	0.171	0.017	0.099
Saguinus imperator	0.029	0.007	0.230	0.028	0.006	0.206	0.190	0.017	0.087	1.134	0.008	0.007	1.061	0.002	0.002	2.162	0.038	0.018	0.448	0.055	0.124	0.256	0.025	0.096
Saguinus midas	0.025	0.002	0.097	0.024	0.003	0.111	0.200	0.022	0.110	1.147	0.006	0.005	1.062	0.002	0.002	2.060	0.093	0.045	0.502	0.009	0.019	0.218	0.024	0.111

**Tibia proximal**	NodDen_mean			NodDen_median			TrabLen			TrabTort_mean			TrabTort_median			FD			DA			BV/TV		
	$\bar{x}$	SD	CV	$\bar{x}$	SD	CV	$\bar{x}$	SD	CV	$\bar{x}$	SD	CV	$\bar{x}$	SD	CV	$\bar{x}$	SD	CV	$\bar{x}$	SD	CV	$\bar{x}$	SD	CV
Leontocebus nigrifrons	0.015	0.002	0.140	0.014	0.001	0.069	0.190	0.016	0.082	1.129	0.019	0.017	1.061	0.004	0.004	2.148	0.050	0.023	0.376	0.008	0.020	0.170	0.055	0.325
Saguinus mystax	0.016	0.002	0.123	0.015	0.002	0.106	0.222	0.006	0.029	1.120	0.009	0.008	1.061	0.001	0.001	2.117	0.037	0.018	0.432	0.068	0.159	0.218	0.021	0.097
Saguinus imperator	0.013	0.004	0.305	0.012	0.003	0.225	0.191	0.037	0.191	1.109	0.009	0.008	1.057	0.005	0.005	2.228	0.065	0.029	0.385	0.083	0.217	0.222	0.036	0.161

**Tibia distal**	NodDen_mean			NodDen_median			TrabLen			TrabTort_mean			TrabTort_median			FD			DA			BV/TV		
	$\bar{x}$	SD	CV	$\bar{x}$	SD	CV	$\bar{x}$	SD	CV	$\bar{x}$	SD	CV	$\bar{x}$	SD	CV	$\bar{x}$	SD	CV	$\bar{x}$	SD	CV	$\bar{x}$	SD	CV
Leontocebus nigrifrons	0.039	0.004	0.102	0.036	0.004	0.113	0.183	0.004	0.021	1.130	0.006	0.005	1.059	0.002	0.002	2.078	0.038	0.018	0.19	0.09	0.50	0.19	0.07	0.34
Saguinus mystax	0.051	0.016	0.313	0.046	0.013	0.287	0.188	0.004	0.019	1.130	0.012	0.011	1.058	0.002	0.002	1.994	0.093	0.047	0.26	0.14	0.53	0.15	0.07	0.46
Saguinus imperator	0.027	0.005	0.185	0.026	0.005	0.185	0.184	0.023	0.127	1.127	0.008	0.007	1.059	0.003	0.003	2.161	0.069	0.032	0.24	0.10	0.44	0.23	0.06	0.27

Note S4: The variation of the trabecular variables studied in this work is shown for the sampled specimens divided by sex, through PCAs performed at each humeral/tibial epiphysis. On PC1-PC2 and PC2-PC3 biplots (shown below):

- A trend of relative increase in FD for female individuals can be detected in the proximal humerus (as one can evince from female individuals tending to occupy the left region of the PC2-PC3 morphospace, together with the respective variable loading plot), distal humerus (as one can evince from female individuals tending to occupy the left region of the PC1-PC2 morphospace, together with the respective variable loading plot), proximal tibia (as one can evince from female individuals tending to occupy the right region of the PC1-PC2 morphospace, together with the respective variable loading plot) and distal tibia (as one can evince from female individuals tending to occupy the left region of the PC1-PC2 morphospace, together with the respective variable loading plot)

- A trend of relative increase in TrabLen for male individuals can be detected in the proximal humerus (as one can evince from male individuals tending to occupy the upper and right regions of the PC1-PC2 and PC2-PC3 morphospaces, respectively, together with the respective variable loading plots)

- A trend of relative increase in BV/TV for female individuals can be detected in the proximal tibia (as one can evince from female individuals tending to occupy the right region of the PC1-PC2 morphospace, together with the respective variable loading plot) and distal tibia (as one can evince from female individuals tending to occupy the left region of the PC1-PC2 morphospace, together with the respective variable loading plot)

- A trend of relative increase in DA for male individuals can be detected in the proximal humerus (as one can evince from male individuals tending to occupy the upper and right regions of the PC1-PC2 and PC2-PC3 morphospaces, together with the respective variable loading plots) proximal tibia (as one can evince from male individuals tending to occupy the left region of the PC1-PC2 morphospace, together with the respective variable loading plot) and distal tibia (as one can evince from male individuals tending to occupy the right region of the PC1-PC2 morphospace, together with the respective variable loading plot)

- A trend of relative increase in TrabTort for male individuals can be detected in the proximal tibia (as one can evince from male individuals tending to occupy the left region of the PC1-PC2 morphospace, together with the respective variable loading plot) and partially, i.e. mainly through TrabTortMean, in the distal tibia (as one can evince from male individuals tending to occupy the right region of the PC1-PC2 morphospace, together with the respective variable loading plot)

Note S4

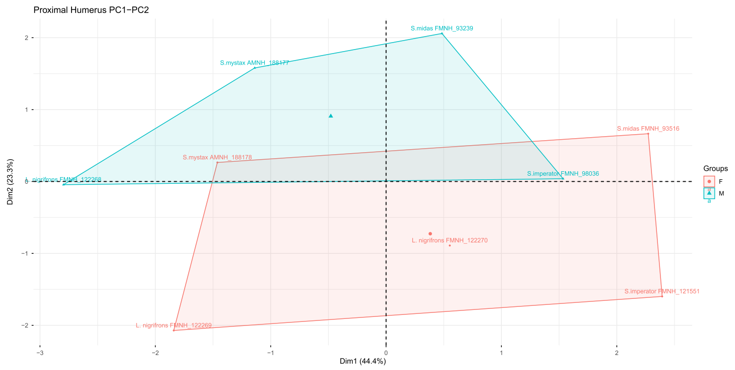
Proximal Humerus PC1−PC2
Dim1 (44.4%)
Dim2 (23.3%)
Groups
F
M

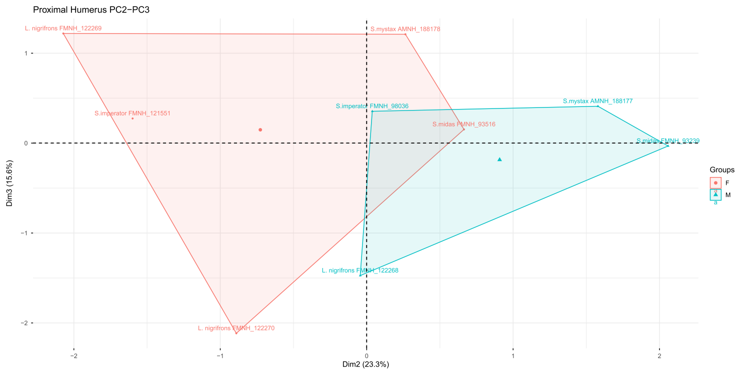
Proximal Humerus PC2−PC3
Dim2 (23.3%)
Dim3 (15.6%)
Groups
F
M

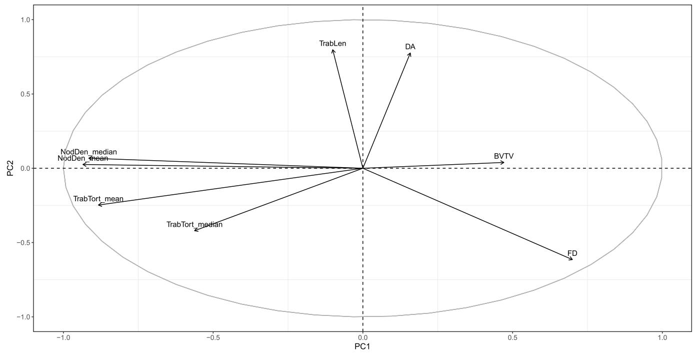
TrabLen
DA
NodDen_median
NodDen_mean
BVTV
TrabTort_mean
TrabTort_median
FD
PC1
PC2

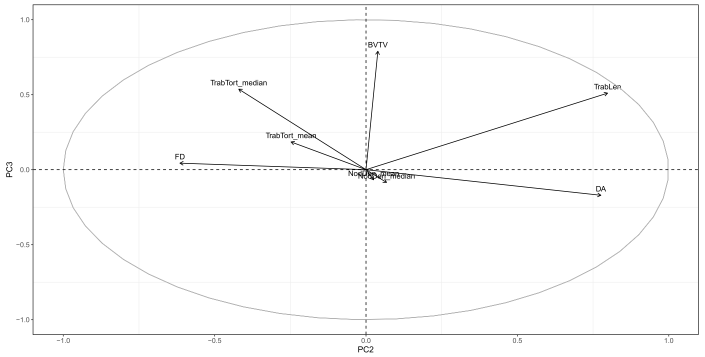
BVTV
TrabTort_median
TrabLen
TrabTort_mean
FD
DA
PC2
PC3

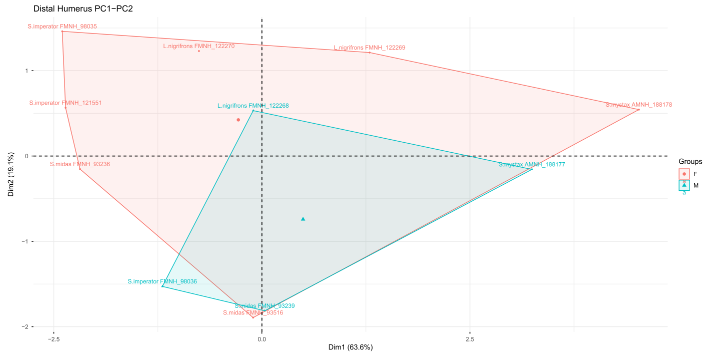
Distal Humerus PC1−PC2
Dim1 (63.6%)
Dim2 (19.1%)
Groups
F
M

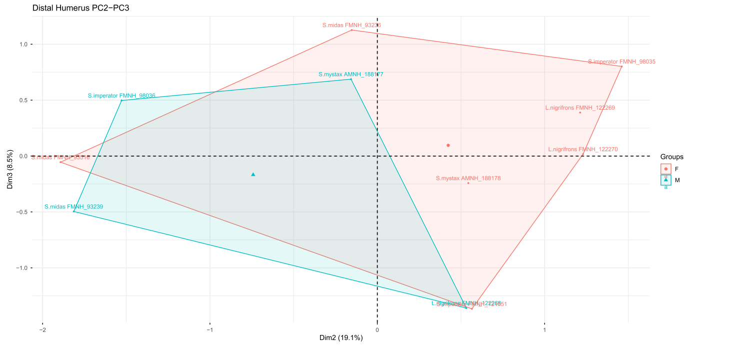
Distal Humerus PC2−PC3
Dim2 (19.1%)
Dim3 (8.5%)
Groups
F
M

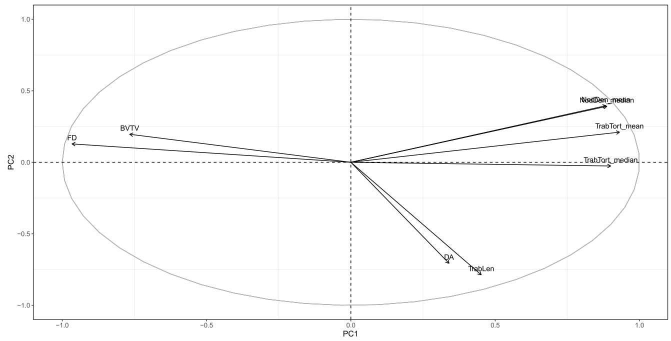
TrabTort_mean
TrabTort_median
BVTV
FD
DA
TrabLen
PC1
PC2

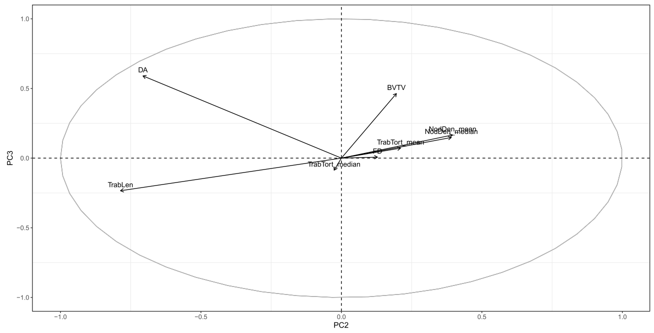
DA
BVTV
NodDen_mean
NodDen_median
TrabTort_mean
FD
TrabTort_median
TrabLen
PC2
PC3

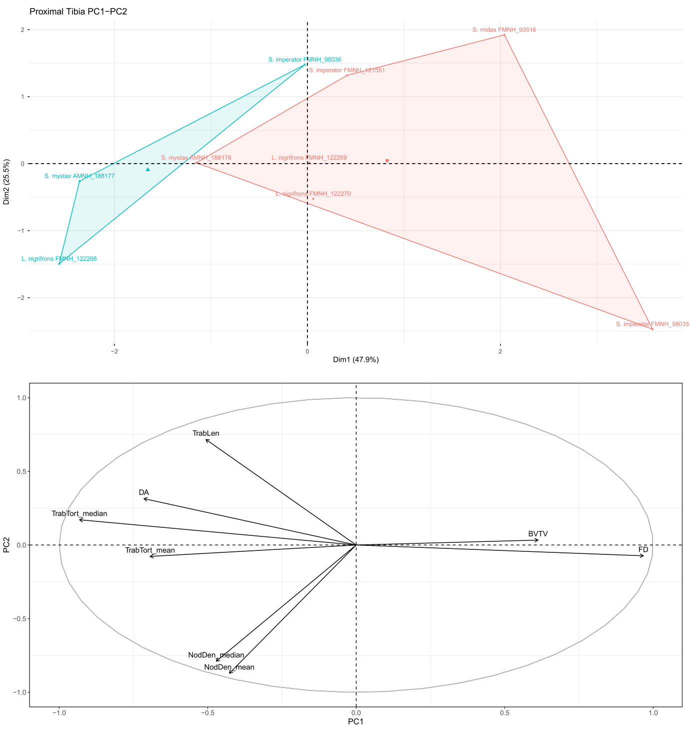
Proximal Tibia PC1−PC2
Dim1 (47.9%)
Dim2 (25.5%)
S. midas FMNH_93516
S. imperator FMNH_98036
S. imperator FMNH_121551
S. mystax AMNH_188178
L. nigrifrons FMNH_122269
S. mystax AMNH_188177
L. nigrifrons FMNH_122270
L. nigrifrons FMNH_122268
S. imperator FMNH_98035
Groups
F
M
TrabLen
DA
TrabTort_median
TrabTort_mean
BVTV
FD
NodDen_median
NodDen_mean
PC1
PC2

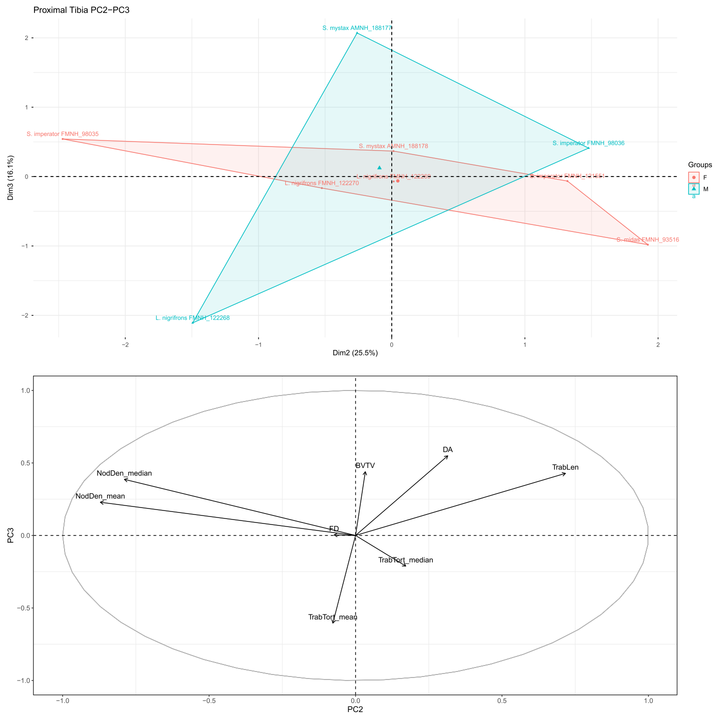
Proximal Tibia PC2−PC3
Dim2 (25.5%)
Dim3 (16.1%)
S. mystax AMNH_188177
S. imperator FMNH_98035
S. mystax AMNH_188178
S. imperator FMNH_98036
L. nigrifrons FMNH_122270
S. midas FMNH_93516
L. nigrifrons FMNH_122268
Groups
F
M
DA
BVTV
TrabLen
NodDen_median
NodDen_mean
FD
TrabTort_median
TrabTort_mean
PC2
PC3

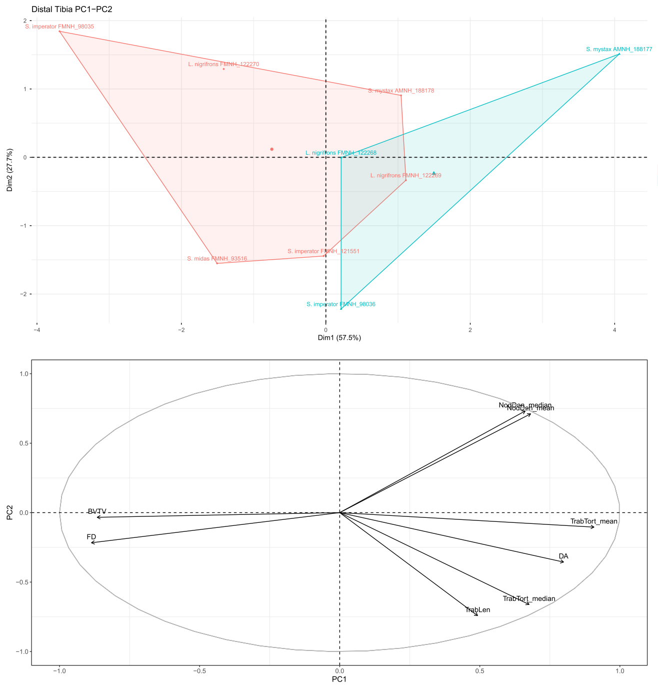
Distal Tibia PC1−PC2
Dim1 (57.5%)
Dim2 (27.7%)
S. imperator FMNH_98035
S. mystax AMNH_188177
L. nigrifrons FMNH_122270
S. mystax AMNH_188178
L. nigrifrons FMNH_122268
L. nigrifrons FMNH_122269
S. midas FMNH_93516
S. imperator FMNH_121551
S. imperator FMNH_98036
Groups
F
M
NodDen_median
NodDen_mean
BVTV
FD
TrabTort_mean
DA
TrabTort_median
TrabLen
PC1
PC2

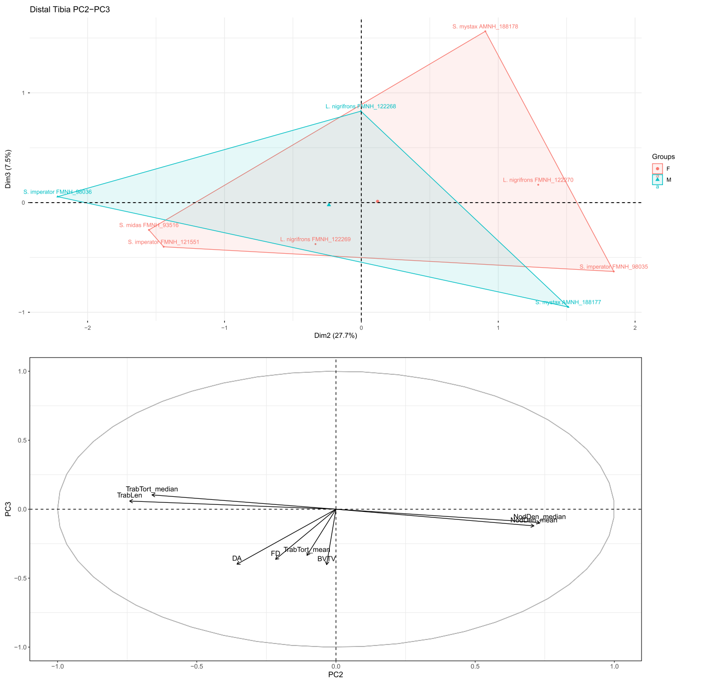
Distal Tibia PC2−PC3
Dim2 (27.7%)
Dim3 (7.5%)
S. mystax AMNH_188178
L. nigrifrons FMNH_122268
L. nigrifrons FMNH_122270
S. imperator FMNH_98036
S. midas FMNH_93516
S. imperator FMNH_121551
L. nigrifrons FMNH_122269
S. imperator FMNH_98035
S. mystax AMNH_188177
Groups
F
M
TrabTort_median
TrabLen
NodDen_median
NodDen_mean
DA
FD
TrabTort_mean
BVTV
PC2
PC3

Note S5: The variation of the trabecular variables studied in this work is shown for the sampled specimens divided by captivity, through PCAs performed at each humeral/tibial epiphysis. PC1-PC2 and PC2-PC3 biplots are shown.

- A trend of decrease in TrabLen for captive individuals can be detected in the proximal humerus (as one can evince from captive individuals tending to occupy the bottom region and the left region of the PC1-PC2 and PC2-PC3 morphospaces, respectively, together with the respective variable loading plot) and the distal humerus (as one can evince from captive individuals tending to occupy the left region of the PC2-PC3 morphospace, together with the respective variable loading plot).

- A trend of relative increase in TrabTort for captive individuals can be detected in the proximal tibia (as one can evince from captive individuals that are driven by TrabTort toward the bottom region of the PC2-PC3 morphospace)

- A trend of relative decrease in BV/TV for captive individuals can be detected in the proximal tibia (as one can evince from captive individuals tending to occupy the bottom region in the PC2-PC3 morphospace, together with the respective variable loading plot)

- A trend of relative increase in FD for captive individuals can be detected in the proximal humerus (as one can evince from captive individuals tending to occupy the left region in the PC2-PC3 morphospace, together with the respective variable loading plot)

- A trend of relative decrease in DA for captive individuals can be detected in the proximal humerus (as one can evince from captive individuals tending to occupy the bottom region and the left region of the PC1-PC2 and PC2-PC3 morphospaces, respectively, together with the respective variable loading plot), distal humerus (as one can evince from captive individuals tending to occupy the left region of the PC2-PC3 morphospace, together with the respective variable loading plot).

Note S5

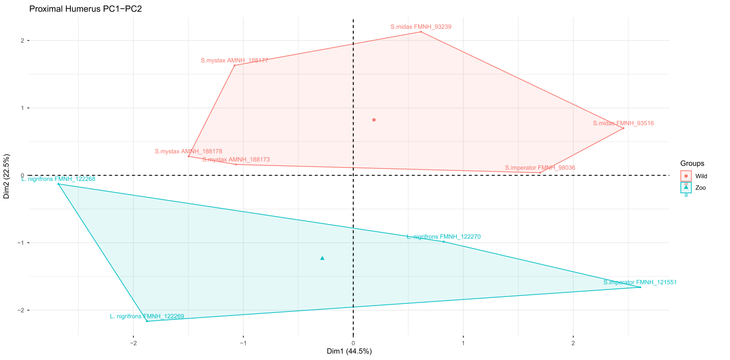
Proximal Humerus PC1−PC2
Dim1 (44.5%)
Dim2 (22.5%)
Groups
Wild
Zoo

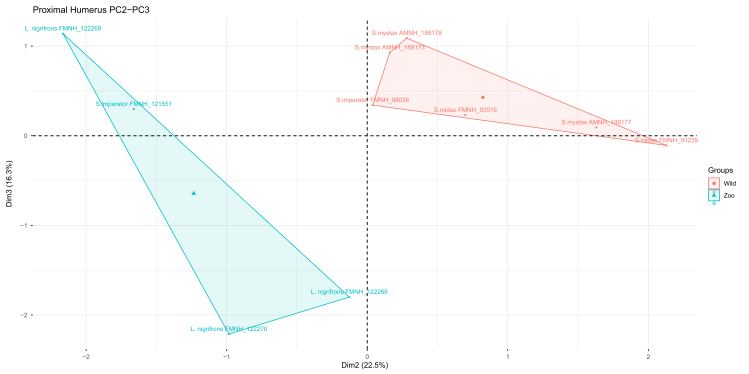
Proximal Humerus PC2−PC3
Dim2 (22.5%)
Dim3 (16.3%)
Groups
Wild
Zoo

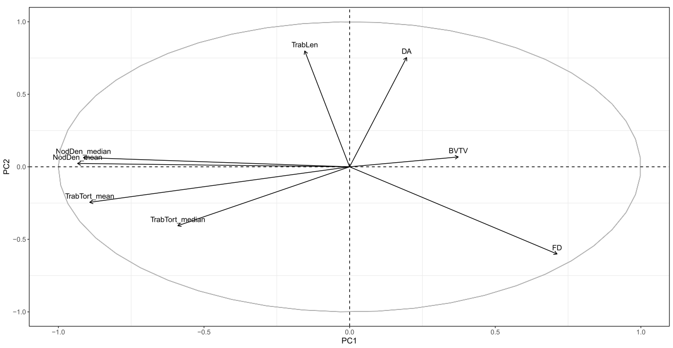
TrabLen
DA
NodDen_median
NodDen_mean
BVTV
TrabTort_mean
TrabTort_median
FD
PC1
PC2

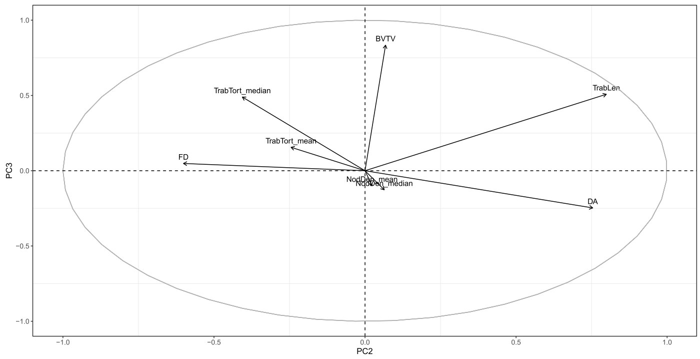
BVTV
TrabTort_median
TrabLen
TrabTort_mean
FD
DA
PC2
PC3

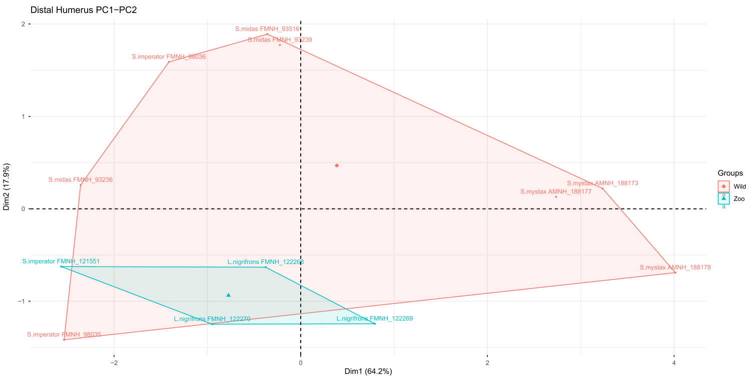
Distal Humerus PC1−PC2
Dim1 (64.2%)
Dim2 (17.9%)
Groups
Wild
Zoo

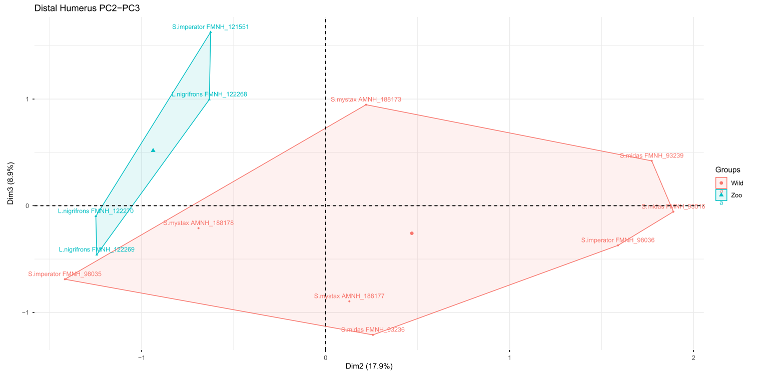
Distal Humerus PC2−PC3
Dim2 (17.9%)
Dim3 (8.9%)
Groups
Wild
Zoo

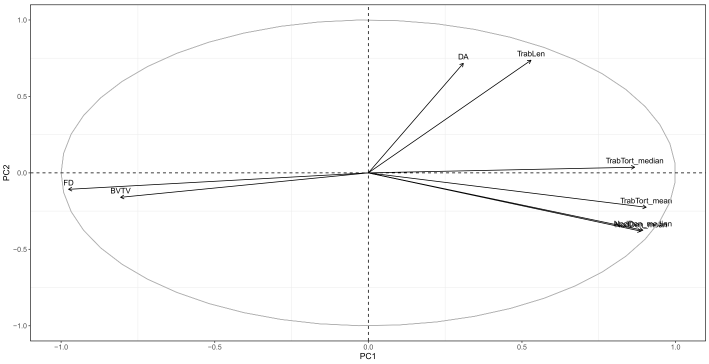
DA
TrabLen
TrabTort_median
FD
BVTV
TrabTort_mean
PC1
PC2

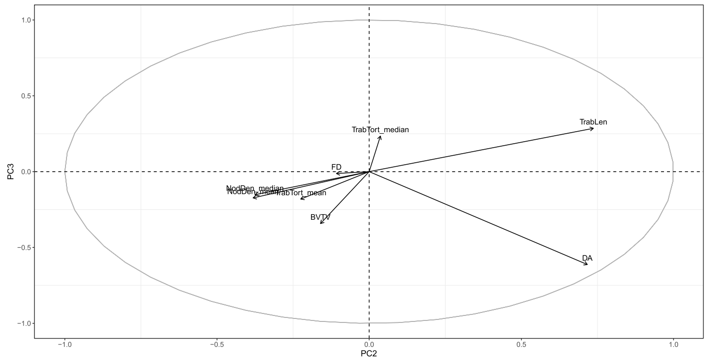
TrabLen
TrabTort_median
FD
BVTV
DA
PC2
PC3

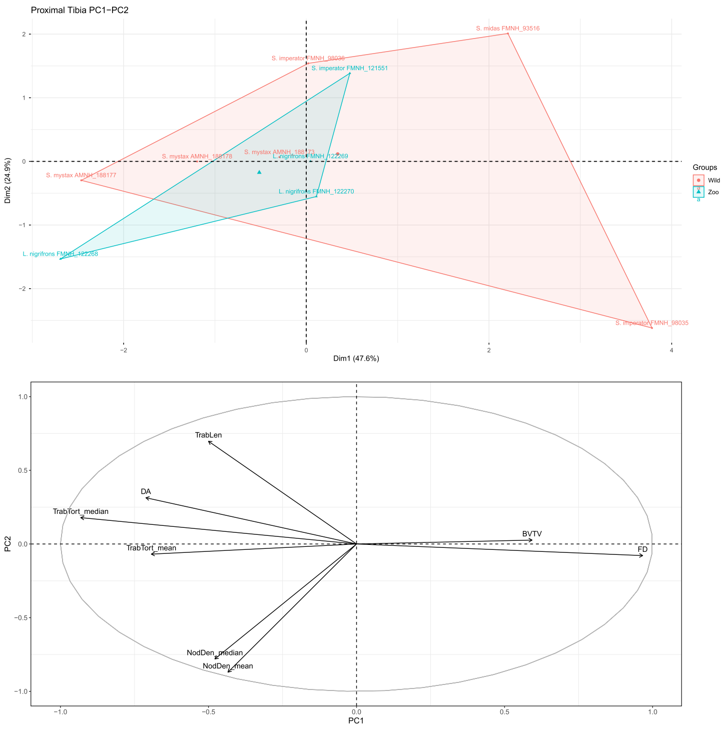
Proximal Tibia PC1−PC2
S. midas FMNH_93516
S. imperator FMNH_98036
S. imperator FMNH_121551
S. mystax AMNH_188178
S. mystax AMNH_188173
L. nigrifrons FMNH_122269
S. mystax AMNH_188177
L. nigrifrons FMNH_122270
L. nigrifrons FMNH_122268
S. imperator FMNH_98035
Dim1 (47.6%)
Dim2 (24.9%)
Groups
Wild
Zoo
TrabLen
DA
TrabTort_median
TrabTort_mean
BVTV
FD
NodDen_median
NodDen_mean
PC1
PC2

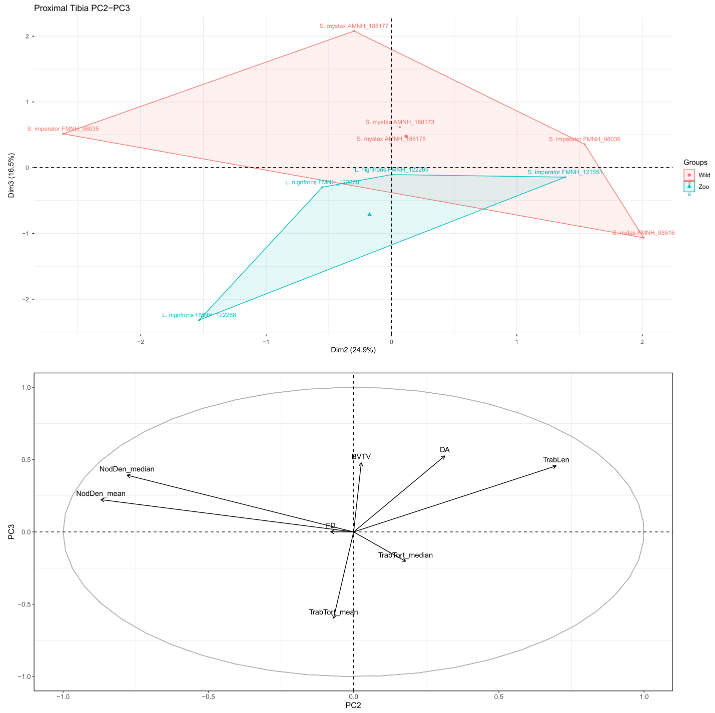
Proximal Tibia PC2−PC3
S. mystax AMNH_188177
S. imperator FMNH_98035
S. mystax AMNH_188173
S. mystax AMNH_188178
S. imperator FMNH_98036
L. nigrifrons FMNH_122270
S. imperator FMNH_121551
S. midas FMNH_93516
L. nigrifrons FMNH_122268
Dim2 (24.9%)
Dim3 (16.5%)
Groups
Wild
Zoo
NodDen_median
NodDen_mean
BVTV
DA
TrabLen
FD
TrabTort_median
TrabTort_mean
PC2
PC3

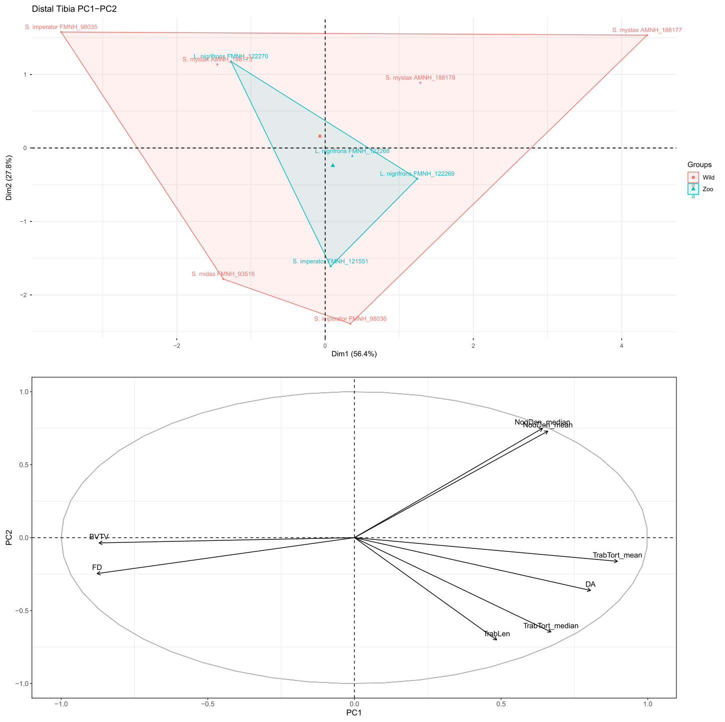
Distal Tibia PC1−PC2
S. imperator FMNH_98035
S. mystax AMNH_188177
L. nigrifrons FMNH_122270
S. mystax AMNH_188173
S. mystax AMNH_188178
L. nigrifrons FMNH_122269
S. imperator FMNH_121551
S. midas FMNH_93516
S. imperator FMNH_98036
Dim1 (56.4%)
Dim2 (27.8%)
Groups
Wild
Zoo
NodDen_median
NodDen_mean
BVTV
FD
TrabTort_mean
DA
TrabTort_median
TrabLen
PC1
PC2

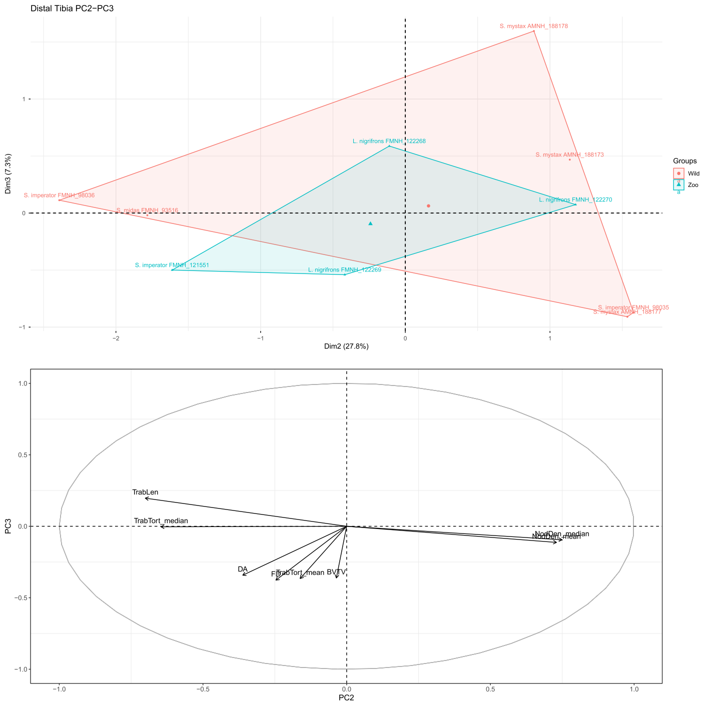
Distal Tibia PC2−PC3
S. mystax AMNH_188178
L. nigrifrons FMNH_122268
S. mystax AMNH_188173
S. imperator FMNH_98036
S. midas FMNH_93516
L. nigrifrons FMNH_122270
S. imperator FMNH_121551
L. nigrifrons FMNH_122269
S. imperator FMNH_98035
S. mystax AMNH_188177
Dim2 (27.8%)
Dim3 (7.3%)
Groups
Wild
Zoo
TrabLen
TrabTort_median
DA
TrabTort_mean
BVTV
NodDen_median
NodDen_mean
PC2
PC3

Note S6-S9: For the proximal humerus (Note S6), distal humerus (Note S7), proximal tibia (Note S8), and distal tibia (Note S9), we generated heatmaps representing the spatial distribution of NodDen values within the epiphyses. To display these, we generated, for each specimen, a series of 2D maps taken at regular spatial intervals corresponding to 10% increments of epiphyseal length. For example, the 20% 2D map shows NodDen values at the level corresponding to 20% of the epiphyseal length, measured from the proximalmost point (see Methods in the main text and Fig. S2 for the definition of epiphyseal length).

In this way, by observing the 2D maps sequentially at 10% intervals proceeding proximodistally, one can appreciate how the distribution of NodDen values varies along the epiphyses. For each map, the colour legend on the right indicates the NodDen values shown in the heatmap. For each specimen, anatomical orientation is specified using four-direction arrows (An: anterior; Po: posterior; La: lateral; Me: medial).

Specimens belonging to long-leaping species are enclosed within a blue rectangle, whereas specimens belonging to short-leaping species are enclosed within a purple rectangle. Green ellipses indicate regions with concentrated high NodDen values, which are particularly common in long leapers. Yellow ellipses indicate epiphyseal levels showing homogeneous NodDen values, that is, a lack of areas with concentrated high values, which are particularly common in short leapers.

Note S6

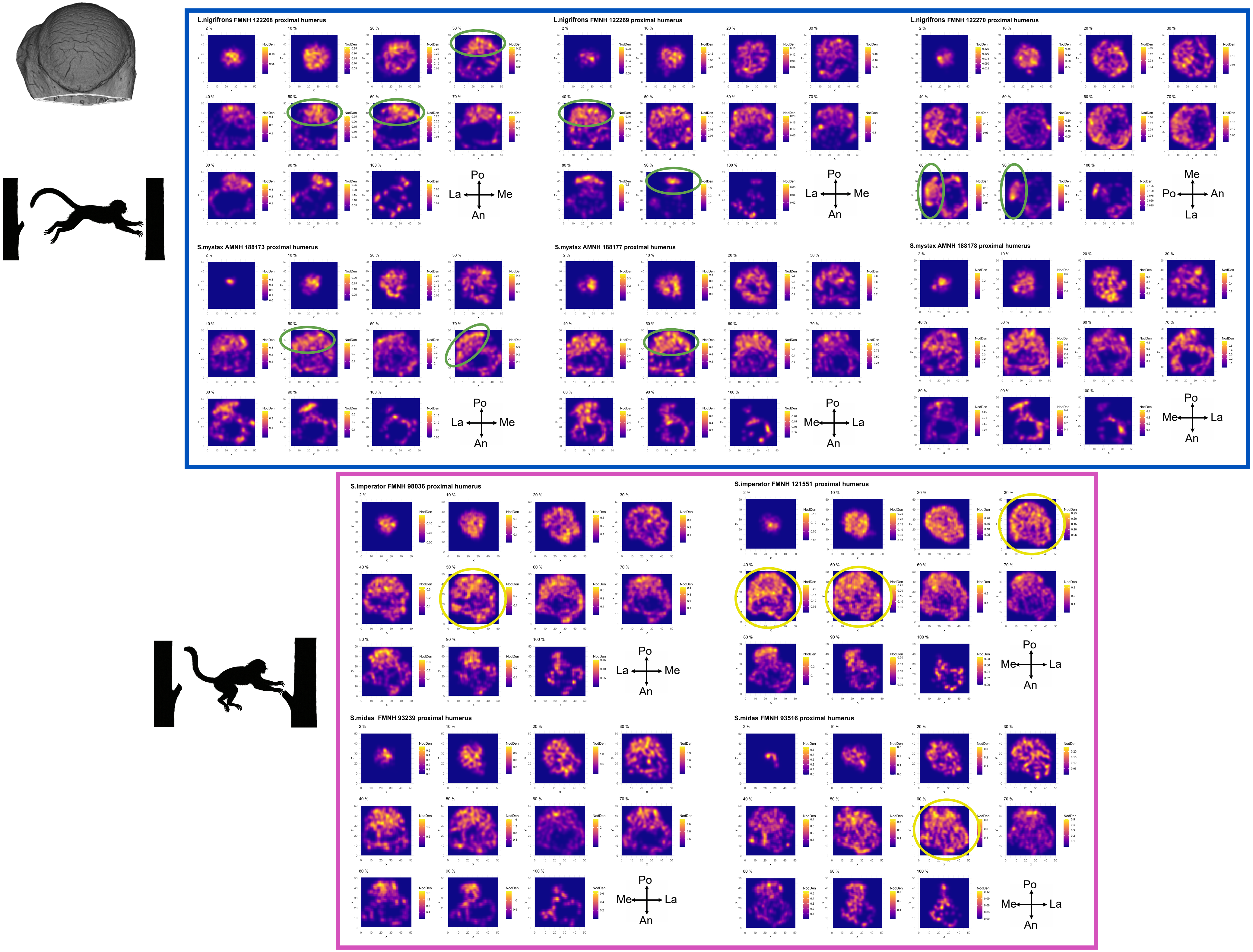

L.nigrifrons FMNH 122268 proximal humerus
L.nigrifrons FMNH 122269 proximal humerus
L.nigrifrons FMNH 122270 proximal humerus
S.mystax AMNH 188173 proximal humerus
S.mystax AMNH 188177 proximal humerus
S.mystax AMNH 188178 proximal humerus
S.imperator FMNH 98036 proximal humerus
S.imperator FMNH 121551 proximal humerus
S.midas FMNH 93239 proximal humerus
S.midas FMNH 93516 proximal humerus
2 %
10 %
20 %
30 %
40 %
50 %
60 %
70 %
80 %
90 %
100 %
NodDen
Po
La
Me
An

Note S7

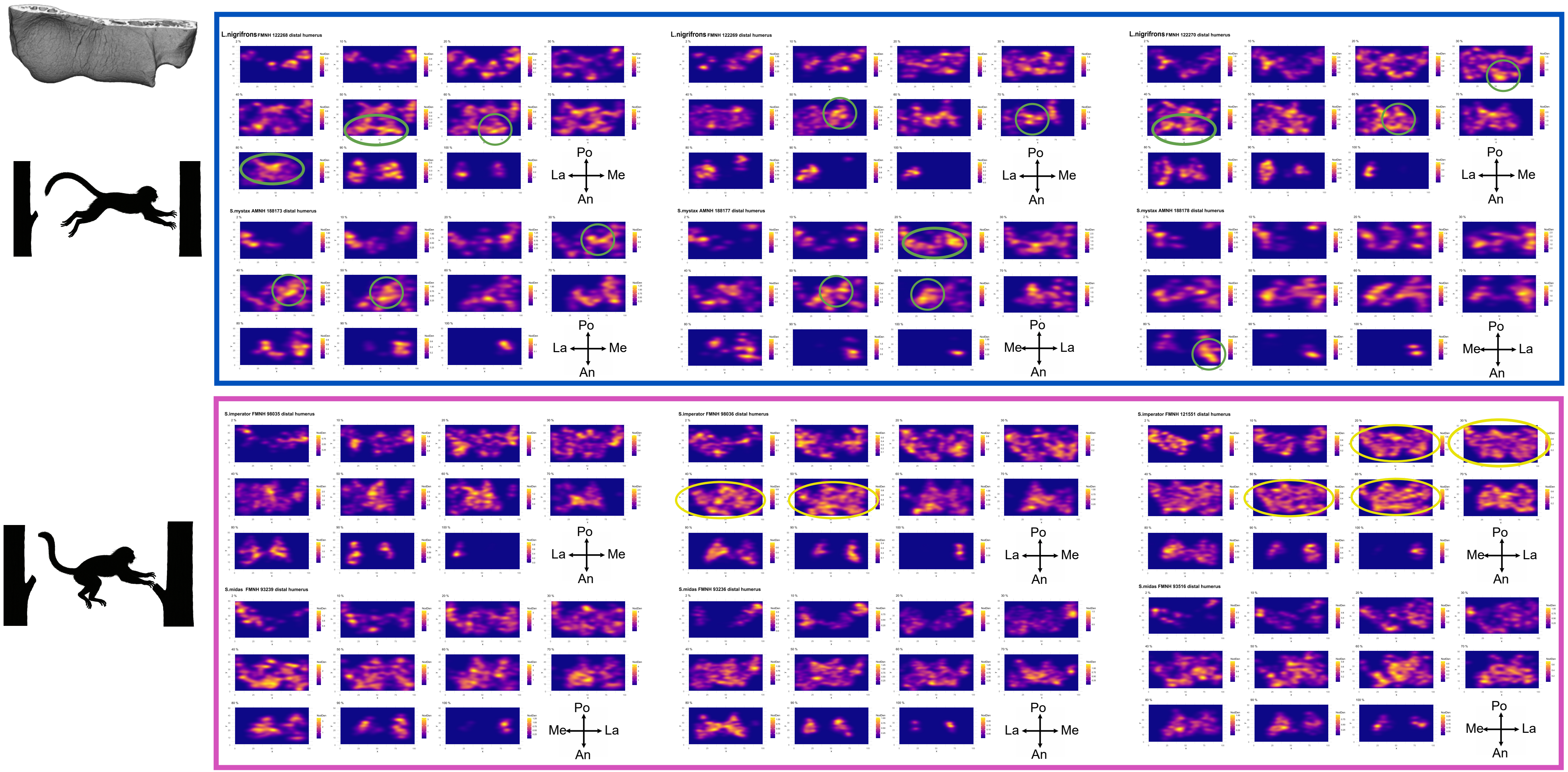

L.nigrifrons FMNH 122268 distal humerus
L.nigrifrons FMNH 122269 distal humerus
L.nigrifrons FMNH 122270 distal humerus
S.mystax AMNH 188173 distal humerus
S.mystax AMNH 188177 distal humerus
S.mystax AMNH 188178 distal humerus
S.imperator FMNH 98035 distal humerus
S.imperator FMNH 98036 distal humerus
S.imperator FMNH 121551 distal humerus
S.midas FMNH 93239 distal humerus
S.midas FMNH 93236 distal humerus
S.midas FMNH 93516 distal humerus
Po
La
Me
An

Note S8

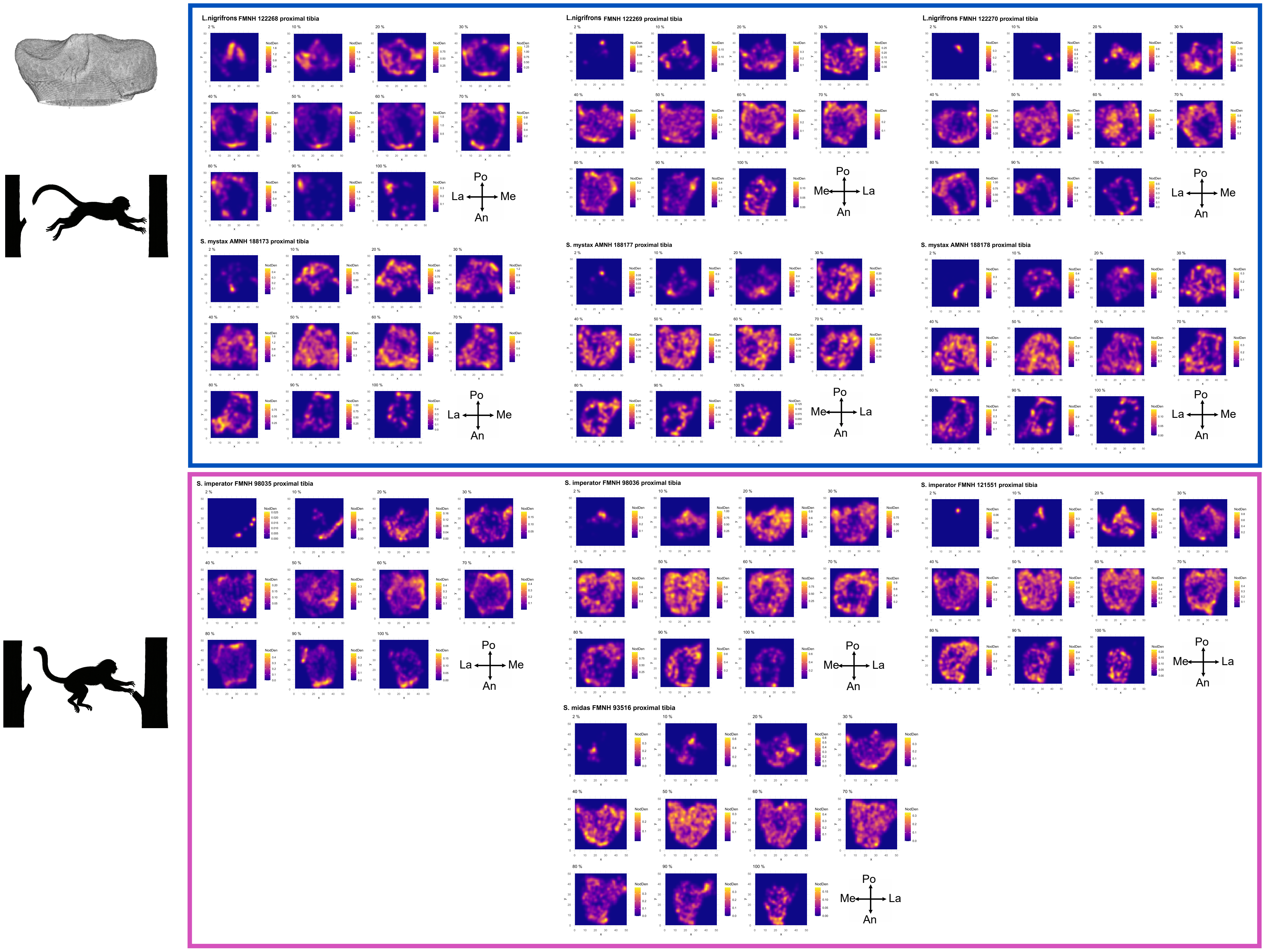
L.nigrifrons FMNH 122268 proximal tibia
L.nigrifrons FMNH 122269 proximal tibia
L.nigrifrons FMNH 122270 proximal tibia
S. mystax AMNH 188173 proximal tibia
S. mystax AMNH 188177 proximal tibia
S. mystax AMNH 188178 proximal tibia
S. imperator FMNH 98035 proximal tibia
S. imperator FMNH 98036 proximal tibia
S. imperator FMNH 121551 proximal tibia
S. midas FMNH 93516 proximal tibia
Po
An
La
Me

Note S9

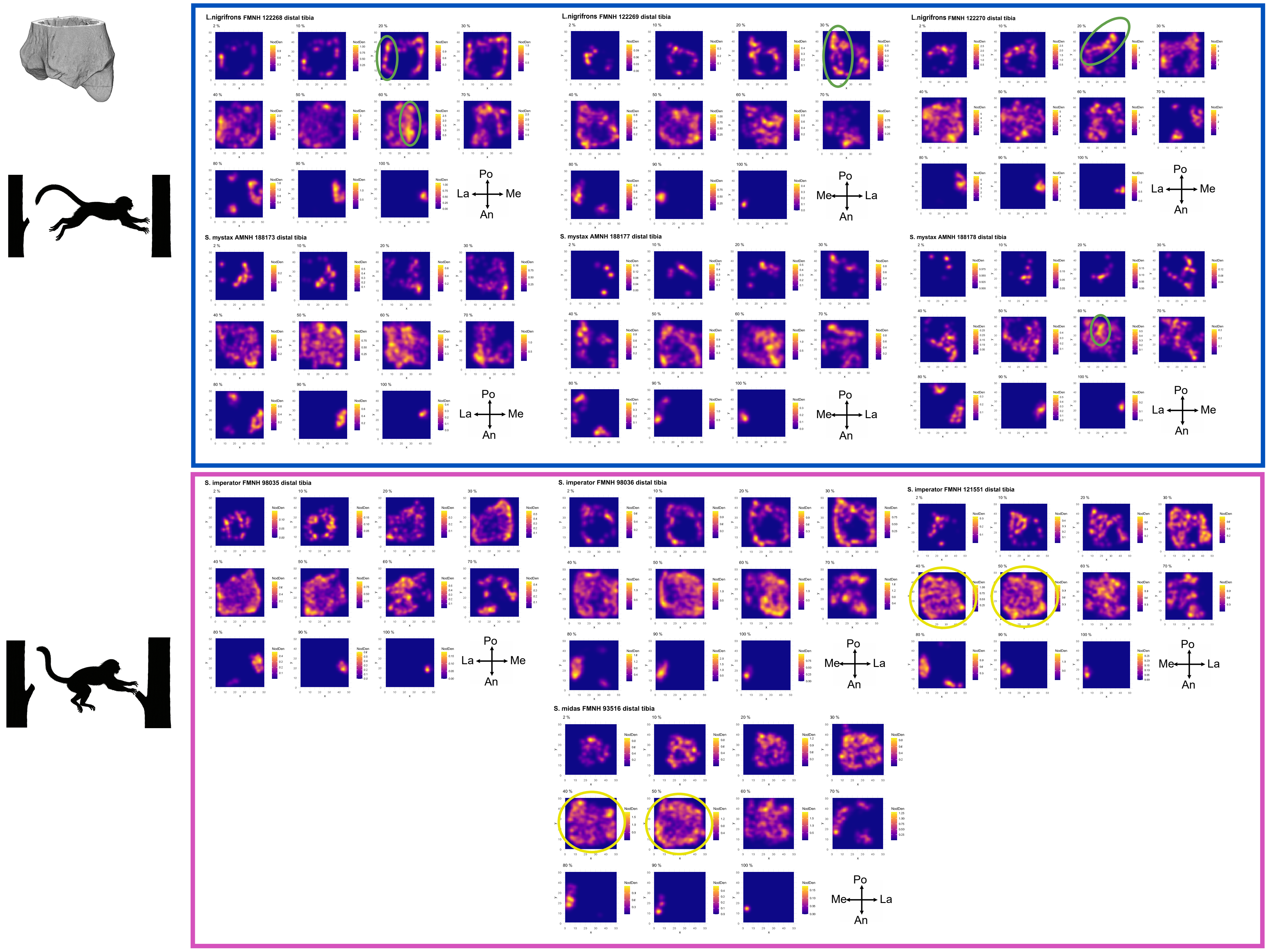

L.nigrifrons FMNH 122268 distal tibia
L.nigrifrons FMNH 122269 distal tibia
L.nigrifrons FMNH 122270 distal tibia
S. mystax AMNH 188173 distal tibia
S. mystax AMNH 188177 distal tibia
S. mystax AMNH 188178 distal tibia
S. imperator FMNH 98035 distal tibia
S. imperator FMNH 98036 distal tibia
S. imperator FMNH 121551 distal tibia
S. midas FMNH 93516 distal tibia
Po
La
Me
An
